# Geographic distribution of the *E1* family of genes and their effects on reproductive timing in soybean

**DOI:** 10.1186/s12870-021-03197-x

**Published:** 2021-09-29

**Authors:** Nicholas Dietz, Rachel Combs-Giroir, Grace Cooper, Minviluz Stacey, Carrie Miranda, Kristin Bilyeu

**Affiliations:** 1 Division of Plant Sciences, University of Missouri, Columbia, MO 65211 USA; 2 Department of Plant Sciences, North Dakota State University, Fargo, ND 58108 USA; 3 USDA/ARS Plant Genetics Research Unit, Columbia, MO 65211 USA

## Abstract

**Background:**

Soybean is an economically important crop which flowers predominantly in response to photoperiod. Several major loci controlling the quantitative trait for reproductive timing have been identified, of which allelic combinations at three of these loci, *E1*, *E2*, and *E3*, are the dominant factors driving time to flower and reproductive period. However, functional genomics studies have identified additional loci which affect reproductive timing, many of which are less understood. A better characterization of these genes will enable fine-tuning of adaptation to various production environments. Two such genes, *E1La* and *E1Lb*, have been implicated in flowering by previous studies, but their effects have yet to be assessed under natural photoperiod regimes.

**Results:**

Natural and induced variants of *E1La* and *E1Lb* were identified and introgressed into lines harboring either *E1* or its early flowering variant, *e1-as*. Lines were evaluated for days to flower and maturity in a Maturity Group (MG) III production environment. These results revealed that variation in *E1La* and *E1Lb* promoted earlier flowering and maturity, with stronger effects in *e1-as* background than in an *E1* background. The geographic distribution of *E1La* alleles among wild and cultivated soybean revealed that natural variation in *E1La* likely contributed to northern expansion of wild soybean, while breeding programs in North America exploited *e1-as* to develop cultivars adapted to northern latitudes.

**Conclusion:**

This research identified novel alleles of the *E1* paralogues, *E1La* and *E1Lb*, which promote flowering and maturity under natural photoperiods. These loci represent sources of genetic variation which have been under-utilized in North American breeding programs to control reproductive timing, and which can be valuable additions to a breeder’s molecular toolbox.

**Supplementary Information:**

The online version contains supplementary material available at 10.1186/s12870-021-03197-x.

## Background

Soybean [*Glycine max* (L.) Merr.] is the world’s most economically important oilseed crop and was domesticated from its wild progenitor (*Glycine soja* [Sieb. & Zucc.]) more than 5000 years ago [1]. It is adapted to temperate latitudes and flowers in response to short day photoperiods. Reproductive timing is critical for optimizing plant yield in any production environment. Several genes to date have been shown to influence flowering and maturity in soybean, including *E1*-*E4* [2–5], *E6-E10* [6–10]*, Tof11* and *Tof12* [11, 12]*,* and *J* [13], while variation in *E1*, *E2*, and *E3* are the predominant loci controlling flowering time in US-adapted varieties [14, 15]. However, further work is needed to identify additional mechanisms useful for fine-tuning reproductive timing in response to photoperiod and to improve adaptation of soybean to various production environments.

The major maturity gene *E1* gene is a B3-related transcription factor which suppresses expression of key florigen genes, *GmFT2a* and *GmFT5a*, in the leaf under long days [16]. The dominant, functional allele, *E1*, delays flowering and maturity by 23 days and 18 days, respectively, compared to the partially functional *e1-as* allele; nonfunctional *e1-fs* and *e1-nl* alleles condition even earlier flowering and may contribute to photoperiod insensitivity [2]. *E2*, an orthologue of the Arabidopsis *GIGANTEA* gene, is a circadian clock gene that plays a role in modulating diurnal expression patterns of floral regulators [3]. The *E3* and *E4* genes are phytochrome molecules involved in perception of red and far-red light, respectively [5]. Phytochrome and circadian clock signals converge to promote transcription of *E1*, and thus inhibit flowering, under long days. This *E1*-mediated repression is relieved once day length shortens past a certain threshold, as determined predominantly by the allelic combination of *E1*, *E2*, and *E3* [16].

Soybean has undergone two whole-genome duplication events in its evolutionary history, with subsequent fractionation back to diploid [17]. As a result, more than 50% of its genes are present as paralogous copies. The major maturity gene *E1* has two paralogues, *E1-like-a* (*E1La*) and *E1-like-b* (*E1Lb*), both of which are located in the pericentromeric region of chromosome 04 [16]. Similar to *E1*, *E1La* and *E1Lb* are transcription factors which exhibit diurnal expression patterns and down-regulate transcription of *GmFT2a* and *GmFT5a* under long day photoperiods [18]. Using incandescent lamps to artificially extend day length, Zhu et al.*,* 2019 showed that *E1Lb* inhibits flowering most strongly under far-red enriched long days, its magnitude similar to that of the *E4* gene [19]. Despite this effect, natural variation in *E1Lb* appears to be very rare among soybean adapted to northern latitudes. Variation in *E1La* has not previously been explored. The gain of photoperiod insensitivity has been categorized into three genotypic groups: 1) disfunction of *e3* and *e4* (*E1/e3/e4*), 2) disfunction of *e1* and *e3* (*e1/e3/E4*), and 3) partial functionality of *E1* with disfunction of *e3* (*e1-as/e3/E4*), in combination with other unknown factors contributing to photoperiod insensitivity [20]. Subsequently, variation in *GmFT5a* [21], as well as disfunction in *e1lb*, have both been implicated in photoperiod insensitivity in an *e1-as/e3/E4* background [19]. Not surprisingly, co-silencing the entire *E1* family of genes in an otherwise extremely late flowering landrace from Southern China led to an apparent complete photoperiod insensitivity under natural daylength and short day conditions [22].

Despite previous reports, the impact that *E1La* and *E1Lb* independently have on flowering and maturity under a natural light regime, as well as the contribution of *E1La* to the expansion of wild and adapted soybean to new production environments, have yet to be explored. In the present study, we show that variation in *E1La* and *E1Lb* each have significant effects on reproductive timing when *E1* is partially functional (*e1-as*), but that the impact of *E1Lb* is abolished in a functional *E1* background. Futhermore, we demonstrate that natural variation in the *E1La* gene has contributed to adaptation of wild soybean to northern latitudes but has been under-utilized as a source of photoperiod insensitivity in cultivars released in North America.

## Results

### Natural and induced variation in the *E1* paralogues, *E1La* and *E1Lb*

Although many genes impacting wild and domesticated soybean phenology have been identified, a subset of flowering time and reproductive period genes are relevant to this research including *E1* and its homologs *E1la* and *E1lb* as well as *Tof11* and *Tof12*, the *GIGANTEA* gene *E2*, and the phytochrome *E3* (Supplemental Table 1) [3, 4, 12, 16, 18]. *E1Lb* was originally positioned on chromosome 18, but the subsequent genome version (Williams 82.a2.v1) has both *E1La* and *E1Lb* positioned on chromosome 04 separated by about 10 million base pairs (Mbp) (Supplemental Table 1). Compared to the characterized variant alleles, the functional versions of *E1*, *E2*, *E3*, *Tof11* and *Tof12* delay flowering and maturity and are the de facto alleles for *G. soja* (Table 1). *Tof11* was not included in the Williams 82 reference genome Williams 82.a2.v1 annotation.

**Table 1:** Maturity gene alleles for soybean parents, controls, and test lines used in population development and characterization

ID	Name	*e1-as*	*E1La*	*E1Lb*	*E2*	*E3*	*Tof11-1*	*Tof12-1*	Experiment^a^
parent	PI 547831	*E1*	*e1la*:K82E	REF	REF	REF	*Tof11*	*Tof12*	A
parent	PI 522226	*E1*	*e1la*:K82E	REF	REF	REF	*Tof11*	*Tof12*	A
parent	KB16-2B#666	*E1/e1-as*	*e1la*:K82E	REF	REF	REF	REF	REF	A
parent	KB16-W2F3	*e1-as*	*e1la*:K82E	REF	REF	REF	REF	REF	A
parent and test	KB17-2#514	*E1*	*e1la*:K82E	REF	REF	REF	REF	REF	A, B
parent and test	KB17-1#481	*e1-as*	*e1la*:K82E	REF	REF	REF	REF	REF	A, B
parent and test	W82 FN	*e1-as*	REF	e1lb:Del	REF	REF	REF	REF	A, B, C
parent and control	Jake	*E1*	REF	REF	REF	REF	REF	REF	A, B, C
control	Ellis HOLL	*E1*	REF	REF	REF	REF	REF	REF	C
parent and control	Williams 82	*e1-as*	REF	REF	REF	REF	REF	REF	A, B, C
parent and control	LG04-6000	*e1-as*	REF	REF	REF	REF	REF	REF	A, B, C
parent	EXP e3	*e1-as*	REF	REF	REF	*e3*	REF	REF	A
control	Brookings	*e1-as*	REF	REF	REF	*e3*	REF	REF	C
parent and control	Deuel	*e1-as*	REF	REF	*e2*	REF	REF	REF	A, C
test	Candor	*e1-as*	REF	REF	*e2*	REF	REF	REF	C

^a^A=breeding; B=2018/2019 field; C=2020 field

**Fig. 1:**
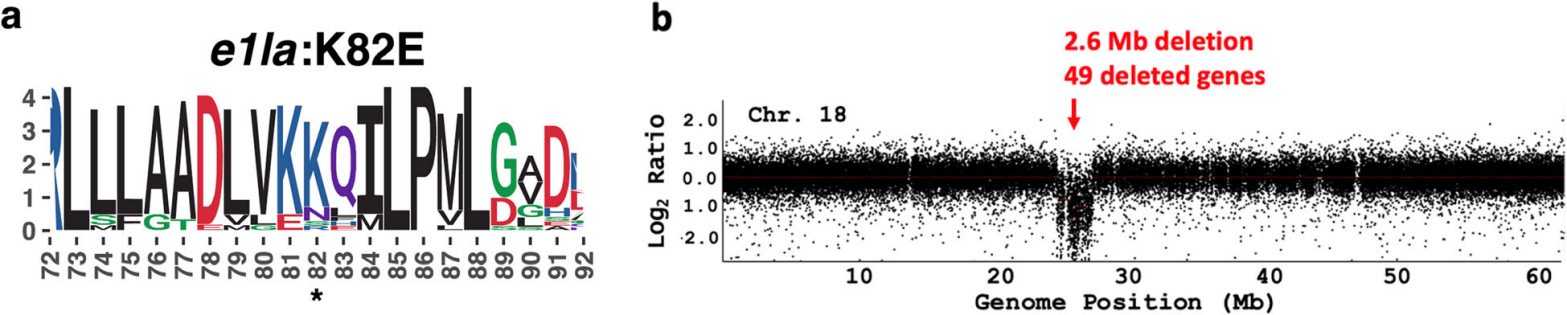
Natural and induced variants of *E1La* and *E1Lb*. **a** Weblogo depicting conserved domain of *E1La* where lysine to glutamate missense change occurs (asterisk). **b** Comparative genomic hybridization depicting deleted region of Chr18 (moved to Chr04 in Wm82.a2.v1) containing the *E1Lb* gene

To determine the potential allelic variation present in *E1La* and *E1Lb*, we conducted a reverse genetics investigation for these genes from among a publicly available set of 302 whole genome re-sequenced accessions containing both *G. max and G. soja* accessions [23]. Using our SNPViz haplotype viewer tool [24], a single nonconservative missense mutation in the *E1La* gene was identified in ten *G. soja* accessions, leading to a lysine to glutamate substitution at amino acid position 82 (hereafter referred to as *e1la*:K82E). The K82E substitution is a positively to negatively charged amino acid change, and it falls within a relatively conserved region of the protein sequence (Fig. 1a; Supplemental Table 2).

Subsequent analysis of an expanded soybean resequencing dataset of 775 accessions distributed between 110 *G. soja* and 665 *G. max* revealed a total of 15 *G. soja* and *2 G. max* accessions with *e1la*:K82E alleles [23, 25]. In our original analysis of the 302 soybean dataset, no variant alleles of the *E1Lb* gene were identified. In a later analysis of the 775 accessions data for the *E1Lb* gene, there were two *G. soja* accessions predicted to contain an S34R missense mutation (data not shown).

We utilized a reverse genetics approach to identify an induced mutant line with a ~ 2.6 Mbp deletion on chromosome 04 that included the *E1Lb* gene from a collection of Williams 82 fast-neutron mutant lines [26]. The boundaries of this lesion were approximated using comparative genomic hybridization (CGH), revealing a deletion of 49 predicted genes in the *G. max* (v1) reference genome, including the *E1Lb* gene (hereafter referred to as *e1lb*:Del) (Fig. 1b; Supplemental Table 3).

### Molecular breeding scheme to develop soybean germplasm with variant alleles of *E1La* and *E1Lb*

To directly investigate the impact *E1La* and *E1Lb* have on flowering time and maturity under natural light conditions, we developed and utilized lines selected by genotype from populations that had segregated for *e1la*:K82E or *e1lb*:Del. New molecular marker assays were developed to track the *e1la*:K82E and *e1lb*:Del mutant alleles. Because undomesticated *G. soja* was the initial source of the *e1la*:K82E alleles, a breeding scheme was devised to isolate those alleles from the confounding effects of *Tof11* and *Tof12* as well as other undesirable *G. soja* agronomic alleles (Supplemental Figures 1 & 2; Table 1). Seven populations were eventually utilized to develop lines with *e1la*:K82E alleles and other combinations of *E1*, *E2*, and *E3* alleles, while two populations were used to develop lines with *e1lb*:Del alleles and either *E1* or *e1-as* alleles (Tables 1 and 2).

**Table 2:** Soybean information for populations developed to select lines for characterization of the *E1La* and *E1Lb* impact on flowering time and maturity

Population	Code	Name	Female	Male	Target 1	Target 2	*G. soja* %	Years	Experiment
1	E1_e1la	KB17-2	Jake	KB16-2B#666	e1la:K82E	*E1*	12.5	2018, 2019	A, B
2	e1_e1la	KB17-1	W82	KB16-2B#666	e1la:K82E	*e1-as*	12.5	2018, 2019	A, B
3	E1_e1lb	KB17-6	W82 FN	Jake	e1lb:Del	*E1*	0	2018, 2019	A, B
4	e1_e1lb	KB16-5	LG04-6000	W82 FN	e1lb:Del	*e1-as*	0	2018, 2019	A, B
5	e1la_e2	KB18-1	Deuel	KB17-1#481	e1la:K82E	*e2*	6.3	2020	C
6	e1la_e3	KB18-23	EXP e3	KB17-1#481	e1la:K82E	*e3*	6.3	2020	C
7	e1_e1la	KB18-16	W82	KB17-1#481	e1la:K82E	*e1-as*	6.3	2020	C
8	e1_e1la	KB18-17	W82	KB16-W2F3	e1la:K82E	*e1-as*	12.5	2020	C
9	E1_e1la	KB18-18	Jake	KB17-2#514	e1la:K82E	*E1*	6.3	2020	C

### Impact of *E1La* and *E1Lb* on reproductive timing in soybean under natural light conditions

**Fig. 2:**
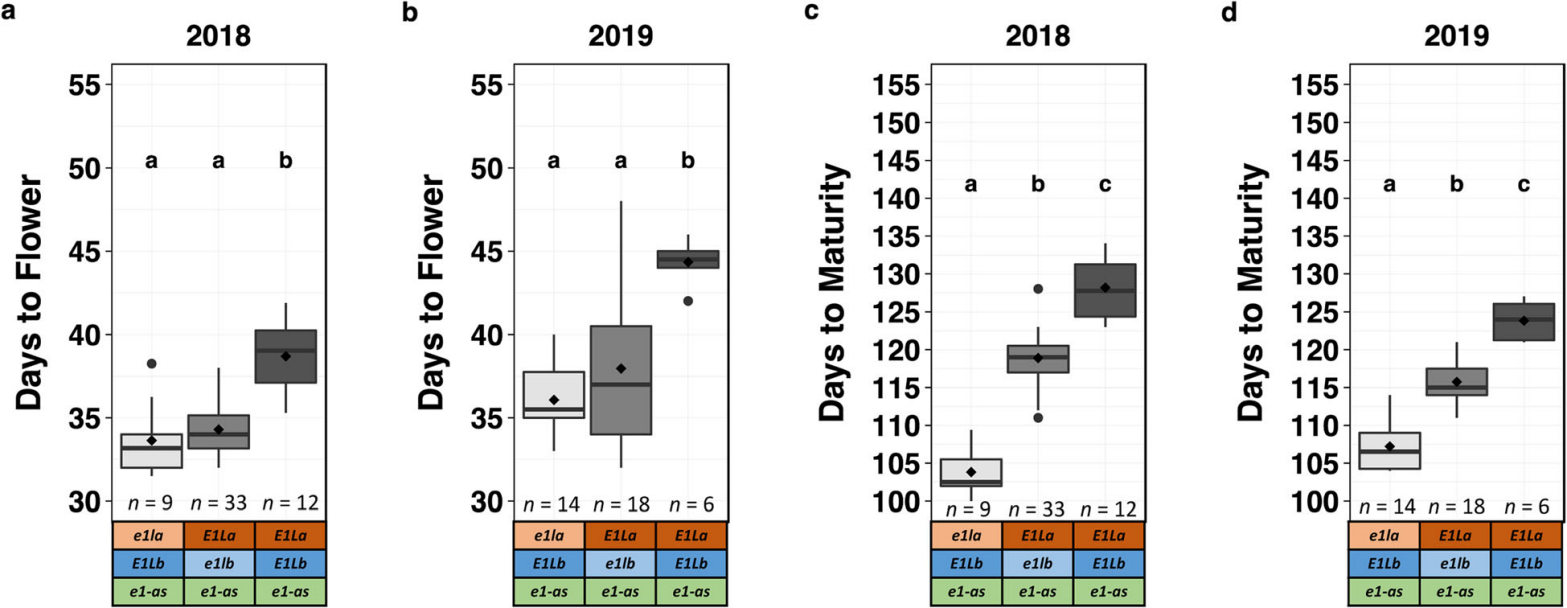
For the *e1-as* background, days to flower and days to maturity for *e1la*:K82E test lines, *e1lb*:Del test lines, and *E1La E1Lb* reference (REF) controls. Means comparisons were conducted using an ANOVA, and significance groups were obtained using a Fisher’s LSD test with false discovery rate (FDR) correction (*P* = 0.05). Within each plot, categories that were not significantly different share the same significance letter. Lines were categorized by their selected genotypes, and *n* represents the number of plots of lines (each replicated three times) per genotype category. *e1la*:K82E is represented as *e1la*, *e1lb*:Del is represented as *e1lb*, and *E1La* and *E1Lb* indicate the REF alleles. Box plots show the mean values (diamonds), median (solid line), quartile span of the data (box), range (vertical lines), and outliers (dots). **a** Days to flower in 2018 experiment; **b** Days to flower in 2019 experiment; **c** Days to maturity in 2018 experiment; **d** Days to flower in 2019 experiment

**Fig. 3:**
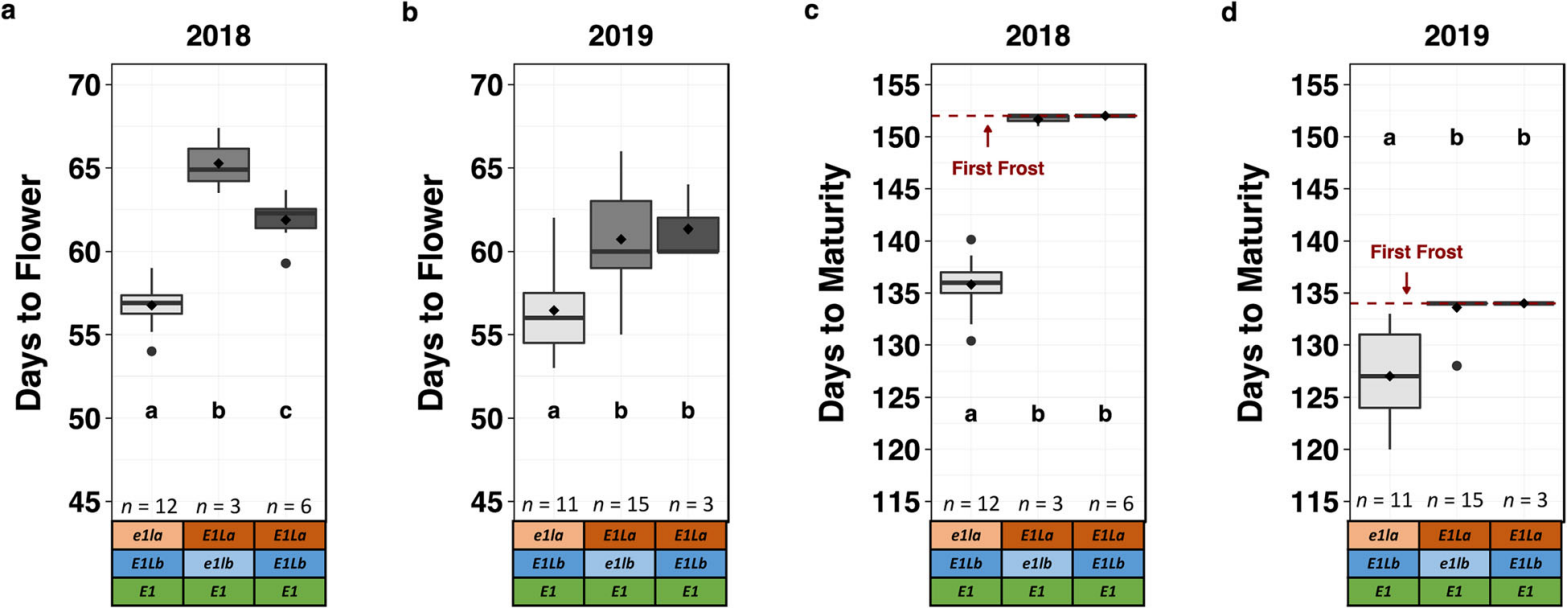
For the *E1* background, days to flower and days to maturity for *e1la*:K82E test lines, *e1lb*:Del test lines, and *E1La E1Lb* reference (REF) controls. Means comparisons were conducted using an ANOVA, and significance groups were obtained using a Fisher’s LSD test with false discovery rate (FDR) correction (*P* = 0.05). Lines were categorized by their selected genotypes, and *n* represents the number of plots of lines (each replicated three times) per genotype category. *e1la*:K82E is represented as *e1la*, *e1lb*:Del is represented as *e1lb;* and *E1La* and *E1Lb* indicate the REF alleles. Box plots show the mean values (diamonds), medians (solid lines), quartile span of the data (boxes), range (vertical lines), and outliers (dots). For days to maturity, the first frost in 2018 (152 days) and 2019 (134 days) is indicated, and all plots not mature at first frost were assigned that value for maturity. **a** Days to flower in 2018 experiment; **b** Days to flower in 2019 experiment; **c** Days to maturity in 2018 experiment; **d** Days to flower in 2019 experiment

Although the *e1la*:K82E alleles have not been previously assessed and *e1lb*:Del alleles were the result of induced mutation, the *e1-as E2 E3 E1La E1Lb* genotype is known to predominate in soybean cultivars adapted to MG III environments in the US [14]. Our adapted reference control line Williams 82 therefore contains the genotype *E1La E1Lb e1-as E2 E3* (Table 1). A subset of the population parents or control lines, and test lines with mutant *E1La* or *E1Lb* were selected from the developed populations (Table 2; Supplemental Table 4) and grown in our MG III Missouri field environment during the 2018 and 2019 growing seasons. Plots were evaluated for phenotypes for days to flower and days to maturity, and there were significant differences for the phenotypes and for the year effect (Figs. 2 and 3). In 2018, lines fixed for *e1la*:K82E/*e1-as* flowered 5 days earlier, and matured 24 days earlier, than lines with reference (REF) alleles for *E1La* and *E1Lb (e1-as)*. Lines fixed for *e1lb*:Del/*e1-as* flowered 4 days earlier, and matured 9 days earlier, than lines with REF allleles for *E1La* and *E1Lb* (*e1-as)*. There was no significant difference between days to flower for *e1la*:K82E and *e1lb*:Del lines, but *e1la*:K82E lines were significantly earlier for days to maturity than *e1lb*:Del lines in the *e1-as* background.

The absolute values were different in 2019 than 2018, but the results were similar; in 2019, lines fixed for *e1la*:K82E/*e1-as* flowered 8 days earlier, and matured 16 days earlier, than lines with reference alleles- *E1La* and *E1Lb* (*e1-as)*. Lines fixed for *e1lb*:Del/*e1-as* flowered 6 days earlier, and matured 8 days earlier, than reference lines- *E1La* and *E1Lb* (*e1-as)* in 2019, and similar to 2018, *e1lb*:Del lines in the *e1-as* background were not significantly different than *e1la*:K82E/*e1-as* for days to flowering, but were significantly later than *e1la*:K82E/*e1-as* lines and earlier than reference lines for days to maturity (Fig. 2).

Soybean lines with functional versions of the *E1* gene are not typically adapted to a MG III field environment [14], but we combined *E1* with the mutant alleles of *E1La* or *E1Lb* (Table 2; Supplemental Table 4) to investigate their ability to influence photoperiod response (Fig. 3). The first frost in a MG III environment typically occurs before *E1* lines have matured. Lines fixed for *e1la*:K82E/*E1* flowered 5 days earlier compared to the reference controls-*E1Lb*/*E1.* Lines with *e1la*:K82E/*E1* matured 16 days before the killing frost, but reference controls- *E1La* and *E1Lb* (*E1)* and lines fixed for *e1lb*:Del/*E1* were killed by frost. The only line fixed for *e1lb*:Del/*E1* was significantly later for days to flowering compared to the reference controls- *E1La* and *E1Lb* (*E1)* in 2018. In 2019, lines fixed for *e1la*:K82E/*E1* flowered 4 days earlier than reference controls-- *E1La* and *E1Lb* (*E1)*, and matured 7 days before the killing frost. Lines fixed for *e1lb*:Del/*E1* flowered the same day as reference controls- *E1La* and *E1Lb* (*E1)*, and did not mature before first frost (Fig. 3).

**Fig. 4:**
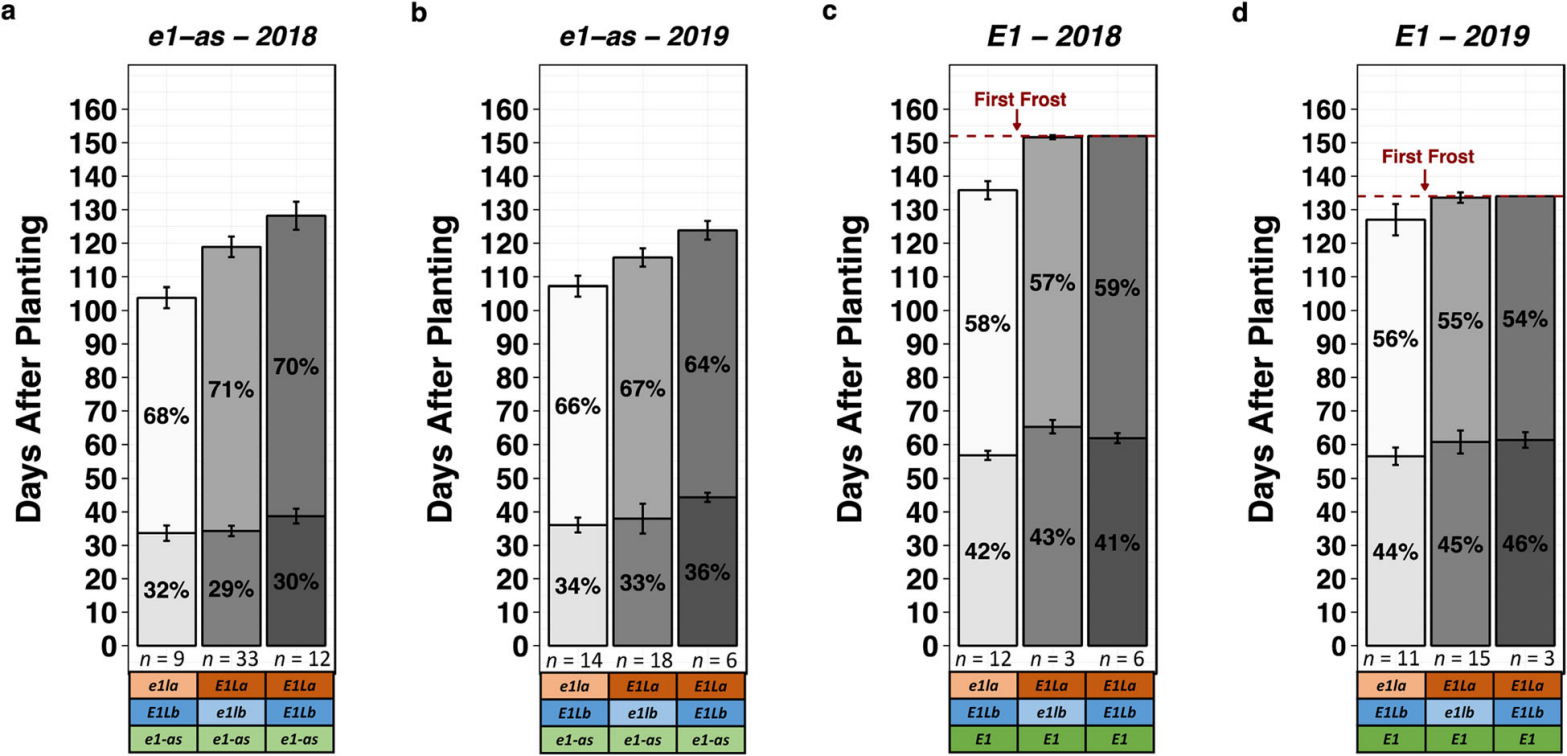
Number of days spent in the vegetative (lower/darker bar) and reproductive phases (upper/lighter bar) for *e1la*:K82E test lines, *e1lb*:Del test lines, and *E1la E1Lb* Reference (REF) controls in both *e1-as* and *E1* backgrounds. Lines were categorized by their selected genotypes, and *n* represents the number of plots of lines (each replicated three times) per genotype category. *e1la*:K82E is represented as *e1la*, *e1lb*:Del is represented as *e1lb*, and *E1La* and *E1Lb* indicate the REF alleles. Bar charts represent the number of days after planting, with the percent of total life span for each phase indicated by (%). Error bars for each group represent the standard deviation. For days to maturity, the first frost in 2018 (152 days) and 2019 (134 days) is indicated, and all plots not mature at first frost were assigned that value for maturity. **a** Length of vegetative and reproductive phases of lines containing *e1-as* evaluated in 2018; **b** Length of vegetative and reproductive phases of lines containing *e1-as* evaluated in 2019; **c** Length of vegetative and reproductive phases of lines containing *E1* evaluated in 2018; **d** Length of vegetative and reproductive phases of lines containing *E1* evaluated in 2019

The length of the reproductive cycle is a critical determinant of plant yield. Given that *E1La* and *E1Lb* pleiotropically affect both flowering time and maturity, we calculated the mean percentage of time spent in each phase of the life cycle (Fig. 4). The relative length of the reproductive phase between lines harboring *e1la*:K82E or *e1lb*:Del, compared to their reference controls, appeared to fluctuate between years. However, in each year, the length of the reproductive phase for genotype groups in the *e1-as* background were always within 3% of its respective control group. It should be noted that parental controls containing the *E1/E1La/E1LB* genotype, and lines containing *E1/E1la/e1lb*:Del, did not mature before the killing frost in either evaluation year. For lines containing these genotypes, the date of the frost was used as the maturation date, and thus the percentages for these genotypes do not represent the true length of the reproductive period. Most interestingly, lines fixed for *e1-as/E1La/E1Lb*, and lines fixed for *E1/e1la*:K82E/*E1Lb*, had significantly different reproductive lengths (average of 67 and 57%, respectively) (Welch’s two-sample t-test, t = 5.83, *p* < 0.001), highlighting a difference in the regulation of reproductive timing between *E1* and *E1La*.

To understand the effects of the *E1La* alleles in a complex maturity background we created soybean lines that were segregating for two additional *E* genes and tested them in our MG III field environment. Further development of soybean germplasm targeted to MG III and MG V but selected for the *e1la*:K82E alleles was done to reduce the *G. soja* genetic background (Supplemental Figures 1 & 2). Soybean lines were developed that combined the *e1la*:K82E alleles with other maturity gene combinations present in MG I (*e1-as e2 E3 E1La E1Lb*) and MG II (*e1-as E2 e3 E1La E1Lb*) soybean varieties (Table 2 and Supplemental Table 4) [14]. A field experiment in our MG III environment for days to flower and days to maturity was conducted in 2020 with the new test lines and parents or controls (Table 3). All lines in the 2020 field experiment had functional *E1Lb* alleles. Similar to the 2018 and 2019 experiment, *e1la*:K82E lines in the MG III background (*e1-as E2 E3 E1Lb*) flowered about 8 days earlier and matured about 10 days earlier than the MG III background control lines (Table 3). The MG I and MG II lines with *e1la*:K82E alleles and either *e2* or *e3* alleles flowered and matured earlier than the control lines for MG I and MG II (Table 3). The new MG V (*E1 E2 E3*) lines fixed for *e1la*:K82E alleles flowered 4 days earlier and matured 8 days earlier than the MG V controls.

**Table 3:** Days to flower and days to maturity and differences for 2020 experimental test lines and their controls

Type	*E* genotype	*E1LA*	n	DTF	ΔDTF^a^	DTM	ΔDTM
MG I	*e1-as****e2****E3*	REF	2	35.5		110.5	
MG I *e1la*:K82E	*e1-as****e2****E3*	*e1la:K82E*	6	33.7	**-1.8**	98.5	**-12**
MG II	*e1-as E2****e3***	REF	1	38.0		115.0	
MG II *e1la*:K82E	*e1-as E2****e3***	*e1la:K82E*	3	34.7	**-3.3**	105.3	**-9.7**
MG III	*e1-as E2 E3*	REF	2	47.0		126.0	
MG III *e1la*:K82E	*e1-as E2 E3*	*e1la:K82E*	10	39.1	**-7.9**	115.6	**-10.4**
MG V	***E1****E2 E3*	REF	2	63.0		140.0	
MG V *e1la*:K82E	***E1****E2 E3*	*e1la:K82E*	2	59.0	**-4**	132.0	**-8**

^a^Difference in days to flower (DTF) or days to maturity (DTM) for lines with contrasting *E1La* genotypes

### Geographic distribution of *E1* and *E1La*

**Fig. 5:**
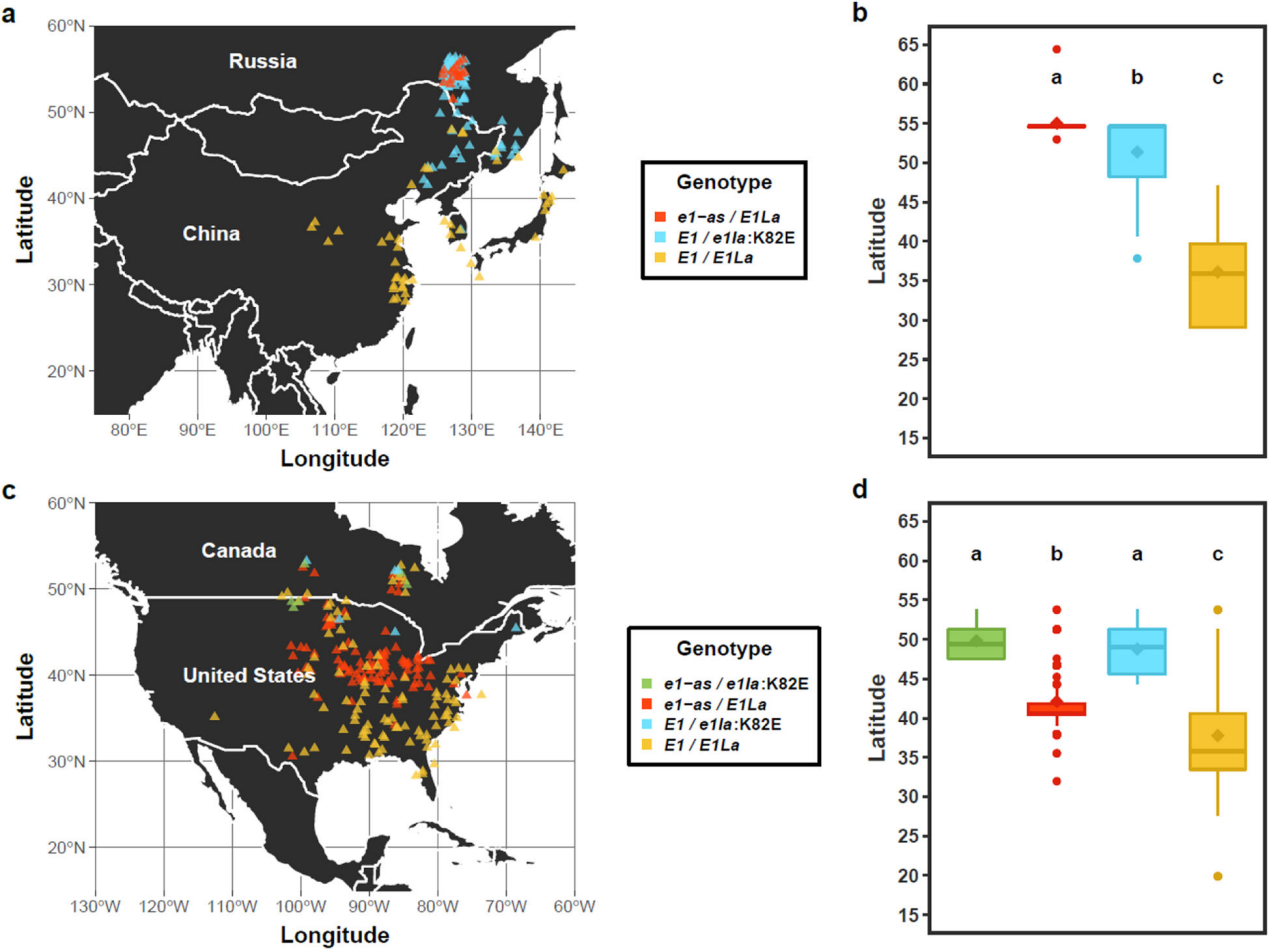
Geographic distribution of *E1* and *E1La* haplotypes. **a**, **b** Subset of 56 *G. soja* accessions from Zhou et al.*,* 2015, plus 92 additional Sanger sequenced *G. soja* accessions (*n* = 148). **c**, **d** North American cultivars present in the GRIN for which *E1* and *E1La* alleles were imputed using SNP50k proxy SNPs (*n* = 592). Means comparisons for boxplots were conducted using an ANOVA, and significance groups were obtained using an LSD test with false discovery rate (FDR) correction (*P* = 0.05). Maps of Asia and North America were obtained using the open-source R package ‘rnaturalearth’

Variant alleles of *E1* and *E1La* are candidates for driving northern expansion of wild soybeans; we hypothesized that the geographic location of *e1-as* and *e1la:K82E* alleles in *G. soja* and *G. max* accessions would illuminate their origin and distribution. For a set of 92 *G. soja* Plant Introduction (PI) accessions from the Germplasm Resources Information Network (GRIN) categorized as Maturity Group II and earlier, we directly determined the allele status of *E1* and *E1La* by Sanger sequencing; in addition, the alleles of *E1* and *E1La* were assigned from re-sequencing data for the subset of 56 *G. soja* accessions from Zhou et al., 2015 for which latitude information could be obtained (Supplemental Table 5). The combined 148 *G. soja* accessions with their *E1* and *E1La* genotypes were assessed for their geographic distribution across soybean’s center of origin in East Asia. The *e1-as* allele was somewhat rare and restricted geographically to far northern regions higher than 50^o^ North latitude (Fig. 5a). The *E1* with *e1la*:K82E allele combination was much more prevalent and spanned a larger latitudinal range, although it was almost entirely absent in accessions originating from below 40°N. Interestingly, northern *G. soja* accessions generally contained either *e1-as* or *e1la*:K82E, but rarely both. Accessions containing the allele combination *E1*/*E1La* had the lowest mean latitude at 36.1°N, while accessions with the allele combinations *E1*/*e1la:*K82E and *e1-*as/*E1La* had higher mean latitudes at 51.4°N and 55°N, respectively (Fig. 5b).

To discern whether the *el1a*:K82E allele has been utilized in North American breeding programs, we conducted an expanded analysis of accessions contained in the GRIN using proxy SNPs from the SoySNP50k array determined to be in high association with either the *e1-as* causative mutation or the *e1la*:K82E causative mutation. The strength of association was estimated using a parameter called “combined pessimistic accuracy,” which is a pairwise calculation between each SoySNP50k marker and the causal mutation that determines the frequency of the Reference and Alternate haplotypes for each position (see Methods for additional details) [14, 27]. The SoySNP50k markers with the highest combined pessimistic accuracy to *e1-as* (ss715593865 – GM06:20916554) and *e1la*:K82E (ss715587601 – GM04:37750626) were used as proxy markers to assess geographic distribution among North American cultivars. The full list of all North American accessions available from the GRIN was filtered to contain only cultivars which had homozygous allele calls for the *E1* and *E1La* proxy SNPs, and for which latitude and longitude information could be obtained. After filtering, the final accession list contained a total of 594 cultivars (Supplemental Table 6). This analysis suggested that the *e1la*:K82E haplotype has likely been used only rarely in North American cultivar development, and that it was exclusive to lines adapted to the northern US and Canada (Fig. 5c). This is in contrast to the *e1-as* allele, which is used extensively in mid and northern MGs throughout the US. Mean latitudes for cultivars with allele combinations *e1-as*/*e1la*:K82E and *E1*/*e1la*:K82e had higher latitudes of 49.8°N and 48.8°N, respectively, when compared to *e1-as*/*E1La* (42.1°N) and *E1*/*E1La*-containing (37.8°N) cultivars (Fig. 5d).

To investigate whether variation in *E1La* may be present in soybean germplasm for which SoySNP50k genotype information was not available, we genotyped *E1La* from among a set of 26 natto and 19 tofu lines from the North Dakota State University breeding program. Interestingly, 24 of the 26 natto lines contained the *e1la*:K82E allele, however, all of the tofu germplasm possessed the Reference allele of *E1La* (Supplemental Tables 7 and 8). This is in contrast to our analysis of the geographic distribution of cultivars using a SoySNP50k proxy SNP, which suggested that the *e1la*:K82E allele was rare in North America. Expanding the use of the Proxy SNP for the *e1la*:K82E allele, we evaluated the frequency of accessions with imputed *e1la*:K82E alleles for *G. soja* and *G. max* GRIN accessions along with their country of origin. For *G. max* accessions, Japan was the origin for 46.8% of the imputed *e1la*:K82E alleles, while the distribution of *e1la*:K82E Proxy SNP was split between Russia, Japan, China, and South Korea for *G. soja* accessions (Supplemental Figure 3).

## Discussion

Soybean is one of the most economically important crops worldwide, with adaptation to the correct photoperiod being critical for adequate yield. Several key genes controlling flowering time and maturity have been cloned and are being utilized extensively in breeding programs. However, many genes have been implicated in reproductive timing in soybean based on functional genomics, but the magnitude of their effects, and their prevalence in breeding programs, are not well understood. Further work is needed to characterize these lesser-known genes before they can be exploited to fine-tune the life cycle of soybean for different production environments.

*E1* and its paralogues *E1La* and *E1Lb* exhibit similar expression patterns, principally a peak just after dawn and just before dusk under long days, and little or no expression under short days [18]. Lines with *E1La* and *E1Lb* down-regulated exhibited higher expression of the florigen promoting genes, *FT2a* and *FT5a*, and earlier flowering than control plants under artificial light, confirming that both functional genes inhibit flowering under long day conditions; however, this study was done in an *e1-nl e2 E3 E4* genetic background [18]. In a study using incandescent lights to extend day length, a single-base deletion mapped to the *E1Lb* gene was shown to confer earlier flowering in a far-eastern Russian cultivar [19]. This *E1Lb* null allele was identified in a total of five Russian soybean cultivars that all had a maturity genotype of *e1-as e2 e3 E4* [19]. RNAi suppression of *E1* and its paralogues resulted in a near-complete loss of photoperiod sensitivity and was sufficient to convert an extremely late-flowering MG VIII cultivar to MG 000 [22]. Our research characterized the role that *E1La* and *E1Lb* each have independently on flowering time and maturity under a natural photoperiod. We identified a lysine to glutamate missense mutation in the *E1La* gene from among a set of publicly available re-sequenced accessions, and identified an induced deletion of the *E1Lb* gene, from which we developed lines in both *e1-as* and *E1* backgrounds. Our results suggest that compared to their variant alleles, functional versions of each of the three members of the *E1* gene family are together contributing to the repression of *FT2a* and *FT5a;* therefore, *E1*, *E1La*, and *E1Lb* suppress soybean flowering and maturity under natural long day photoperiod conditions, consistent with previous gene expression studies [18].

Our field experiments with natural light provided environments that represent soybean production scenarios for maturity group III that are optimized for the the variant *e1-as* alleles along with functional versions of the *E2*, and *E3* genes [14]. The summer solstice at our field location provides 14 h and 54 min of daylight from sunrise to sunset. The daylength typically reaches its maximum and has begun to shorten prior to soybean plants flowering in the field. Taken together, the results demonstrated that, similar to its paralogue *E1*, *E1La* functions to delay flowering and maturity under long day conditions, with the *e1la*:K82E allele having a stronger effect on promoting maturity in an *e1-as* background than in an *E1* background. The *e1la*:K82E alleles also appeared to promote flowering and maturity in genetic backgrounds with additional defects in the major maturity genes *E2* and *E3* when *e1-as* alleles were present. Likewise, the *E1Lb* gene functions to delay flowering and maturity in a partially functional *e1-as* background; however, the ability of *e1lb*:Del to promote flowering appears to be abolished in a fully functional *E1* background. It appears that a natural null allele of *E1Lb* has contributed to adaptation of some soybean cultivars in Russian production environments [19]. While the magnitude of phenotypic effects of the *E1L* genes are different under natural light regimes than artificial light, our results show similar trends to those published in previous reports describing *E1La* and *E1Lb*, with defeciencies in *E1La*, *E1Lb* or both leading to earlier flowering and maturity [18, 19, 22]. Our experiments were intended to provide practical information and novel alleles of new maturity genes that could be used in the context of the established maturity gene combinations to fine-tune the timing of flowering and maturity to optimize photoperiod sensitivity for enhanced yield potential in existing soybean production environments.

In addition, we assessed the role that allelic combinations of *E1* and *E1La* have played in adaptation of wild and cultivated soybean to northern latitudes. This analysis revealed that the *e1la*:K82E allele is present in high frequency in *G. soja* accessions adapted to higher latitudes, and that the *e1-as* allele is relatively rare. An analysis of varieties released in North America using SoySNP50k proxy SNPs suggested a heavy reliance on *e1-as* to develop cultivars adapted to northern production environments, but little use of *e1la*:K82E. However, a direct genotyping analysis of the *E1La* gene in a specialty breeding program in North Dakota revealed that the *e1la*:K82E allele is being exploited to develop natto cultivars. Indeed, there was an apparent high frequency of the *e1la*:K82E allele based on the proxy SNP in wild and cultivated soybeans originating in Japan, where natto is a traditional soyfood [28]. Together, these results revealed that wild soybean and North American breeding programs have exploited different members of the *E1* gene family as the predominant source of reducing photoperiod sensitivity; however, variation in *E1La* may play an important role in adaptation of North American cultivars to far northern latitudes. In concert with the disparity in reproductive lengths we observed between lines fixed for *e1-as* and *e1la*:K82E, this also explains, at least in part, the shorter reproductive phase generally observed in *G. soja* accessions, when compared to *G. max*.

## Conclusions

We identified natural and induced variation in the *E1* paralogues, *E1La* and *E1Lb*, and demonstrated that these variant alleles independently promoted earlier flowering and maturity. Initial efforts suggested that variation in these genes is rare in North American breeding programs, however, further investigation revealed that variation in *E1La* is being exploited in a specialty breeding program in North Dakota. These novel alleles of *E1La* and *E1Lb* constitute valuable resources in a breeder’s toolbox for better adaptation of germplasm to northern production environments.

## Methods

### Natural and induced variation in *E1La* and *E1Lb*

#### E1La

The 302 soybean accessions with whole genome re-sequence data [23] were evaluated in the haplotype visualization tool, SNPViz [24] for variant genomic sequence positions in *E1La* (Glyma04g24640; Wm82.a1.v1.1) on chromosome 04 in the region around position 28,293,933 to 28,294,806, and ten *G. soja* accessions contained a haplotype that included a nonsynonymous A/G variant at position 28,294,378. The ten *G. soja* accessions also contained a synonymous variant (G/T) at Gm04:28,294,356 that was present in an additional 14 *G. soja* accessions. The soybean allele catalog (http://soykb.org/GenescapeAnalysis/search.php) was used to assess the distribution of alleles of *E1La* and *E1Lb* in our curated data set of 775 whole genome re-sequenced soybean accessions [23, 25]. A protein blast at NCBI, using the Williams 82 reference peptide sequence of *E1La*, was used to obtain the orthologous sequences from 23 different legume species with the highest percent identity (Supplemental Table 2). Multiple sequence alignment was generated using the “msa” R package, and the weblogo (trimmed to 20 amino acids) was generated using the “ggplot2” R package.

#### E1Lb

Soybean seeds of cultivar Williams 82 were originally obtained from the GRIN and irradiated with fast neutrons (FN) at 20, 25, 30, and 35 Gy doses at the McClellan Nuclear Radiation Center (University of California, Davis). To determine the copy number variation (CNV) events induced in the mutagenized population, select mutants were analyzed by comparative genomic hybridzation (CGH) using a Roche NimbleGen 696,139-feature soybean CGH microarray following previously published protocol [26, 29]. The oligonucleotide probes of 50- to 70-mers spaced at approximately 1.1 kb intervals were designed based on the Williams 82 genome sequence (Wm82.a1 version). Copy number variation events were called following previously set criteria [29]. Based on the detected CNVs from the CGH analysis, we identified a mutant (MO12) harboring ~ 2.6 Mbp deletion on chromosome 04 encoding the *E1Lb* gene [26].

As part of a separate project, seeds from the entire set of *G. soja* accessions were obtained from the GRIN, and a subset of 419 of the accessions phenotyped for maturity group II or earlier was selected for characterization. DNA was isolated from ground seed tissue using the DNeasy Plant Mini Kit (Qiagen, Inc., Valencia, CA) according to the manufacturer’s instructions. Samples were first evaluated for *E1* or *e1-as* alleles using our established SimpleProbe assay [14], and of those that were successfully genotyped, 57 were *e1-as*, and 244 accessions were *E1*. Subsequently, 84 *E1* and 20 *e1-as* accessions were evaluated for their *E1La* sequences. Sanger sequencing at the University of Missouri DNA Core Facility of 642 bp *E1La* PCR amplicons from *G. soja* accessions utilized PCR primers E1La-F1: 5′- AAACACTCAAAGCCCGATCA-3′ and E1La-R2: 5′- GATTTGAAAGTAGAATAAAGCTAACACAG-3′ as described previously, except amplicons were isolated by ethanol precipitation prior to sequencing with the primer E1La-F1 [30].

##### Molecular marker assays

Plant samples for genotyping were tagged and leaf presses were prepared on FTA cards as described [31]. A new Simple probe genotyping assay was developed to track *E1La* or *e1la*:K82E alleles. Reactions were carried out in 20 μl containing template, primers, 0.2 μM final concentration of SimpleProbe, buffer (40 mM Tricine-KOH [pH 8.0] 16 mM MgCl2, 3.75 μg ml-1 BSA,), 5% DMSO, 200 μM dNTPs, and 0.2X Titanium Taq polymerase (BD Biosciences, Palo Alto, CA). The *e1la*:K82E antisense probe was 5′-Fluorescein-SPC-GGCAAAATTTGCTCCTTCACCAAATC-Phosphate-3′. Originally, the primers E1La-F1 and E1La-R2 were used in the assay, but difficulties amplifying from FTA card samples led to the replacement with nE1La-F1 (5′-GGGAGTTTCAACAACACTGAAGC-3′) and nE1La-R2 (5′-GGTGTCCATGTCCCAAACTCTAAC-3′) that targeted a 233 bp product. In both cases, the forward primers (E1La-F1 or nE1La-F1) were used at 5 μM final concentration and the reverse primers (E1La-R2 or nE1La-R2) were used at 2 μM final concentration in the PCR. Genotyping reactions were performed using a Lightcycler 480 II real time PCR instrument (Roche), using the following PCR parameters: 95 °C for 5 min followed by 40 cycles of 95 °C for 20 s, 60 °C for 20 s, 72 °C for 20 s, and then a melting curve from 50 °C to 70 °C.

A Tm-shift assay [32], based on GC primer tails of differing lengths, was developed to discriminate between *E1Lb* and *e1lb*:Del alleles. Because the *e1lb*:Del allele is a deletion of the entire *E1Lb* gene, this assay was unable to distinguish between the REF *E1Lb* allele and lines that were heterozygous for *E1Lb*. Reactions were carried out in 20 μl containing template, primers, 0.063 μM final concentration of EvaGreen Fluorescent Dye (Biotium, San Francisco, CA), buffer (40 mM Tricine-KOH [pH 8.0] 16 mM MgCl2, 3.75 μg ml-1 BSA), 5% DMSO, 200 μM dNTPs, and 0.2X Titanium Taq polymerase (BD Biosciences, Palo Alto, CA). Genotyping reactions were performed using a Lightcycler 480 II real time PCR instrument (Roche), using the following PCR parameters: 95 °C for 3 min followed by 35 cycles of 95 °C for 20 s, 60 °C for 20 s, 72 °C for 20 s, and then a melting curve from 72 °C to 87 °C. Primers for *E1Lb* were: E1Lb-F1 (5′-GTGTAAACACTCAAAGTCCTT-3′), E1Lb-R (5′-CTCCTCTTCATTTTTGTTGCTGC-3′), 3Ad1 (5′-TTGCATCACCATGGTCATCAT-3′), 3Aix (5′-AGCTATTATCTAGCATTAACCTCA-3′).

Simple probe assays were utilized for genotyping *E1*/*e1-as* and *E2/e2* as previously described [14]; a gel-based assay of PCR products was used to distinguish *E3* from *e3-tr* alleles [24].

The *Tof11*/*tof11–1* and *Tof12*/*tof12–1* allele genotyping assays utilized a GC tail and a nonspecific DNA-binding dye, and produced distinct melting temperatures for different alleles [32]. Genotyping assays were conducted as previously described except EvaGreen (Biotium) was used as the dye at 0.063 μM final concentration [33], and primers for *Tof11* were DTF1tailf1: 5′-GCAACACCTTGACAATCAGAAT-3′, DTF1tailW82r2(5′- gcgggcagggcggcAGCCACATTGCCATTTCTA-3′), and DTF1tailGsr2 (5′- gcgggcAGCCACATTGCCATTTTCAA-3′). Primers for *Tof12* were DTf2TailW82 (5′-gcgggcagggcggcCATAAAGCTGCAGTAGATACCT-3′), DTf2TailGs (5′-gcgggcCATAAAGCTGCAGTAGATTCCC-3′), and DTf2TailR1 (5′-GCATTTGATGATACACATTGCG-3′).

### Development of mutant *e1la* and *e1lb* populations

#### Plant materials

Seeds of *G. soja* accessions PI52226 and PI547831 containing *e1la*:K82E alleles were obtained from the GRIN. A line from fast neutron mutagenesis of Williams 82 was the source of the W82 FN *e1lb*:Del alleles. Jake is a MG V determinate cultivar provided with permission by the developer [34], Williams 82 is a MG III indeterminate cultivar obtained from the GRIN [35], Deuel is a MG I indeterminate cultivar released by South Dakota Agricultural Experiment Station, PVP 201000318 and provided with permission by the developer; Brookings is a late MG I cultivar released by South Dakota Agricultural Experiment Station, and provided with permission by the developer, LG04–6000 is a MG IV indeterminate cultivar provided with permission by the developer [36], Candor is an early MG II cultivar provided with permission from Sevita International, Ellis HOLL is an experimental seed composition MG V determinate line from the University of Tennessee provided with permission by the developer, and the EXP *e3* line was an experimental seed composition line verified to contain *e3-tr* alleles developed by the authors [4, 24]. The genes relevant to this work as parent lines are classified as having the reference Williams 82 alleles (REF) or the indicated alternate alleles specific to each gene (Table 3).

#### Breeding schemes

Soybean populations were developed with different parent combinations (Table 2). Generally, F_1_ seeds were produced at the South Farm Research Center near Columbia, Missouri during the summer field season followed by a cycle of self-pollination that produced F_2_ seeds in the winter nursery near Upala, Costa Rica. During the second cyle in the winter nursery, the F_2_ plants were sampled and underwent genotypic selection prior to single plant harvest of F_2:3_ seeds. To isolate the *e1la*:K82E alleles out of the *G. soja* genetic background, breeding efforts with PI547831 and PI522226 with *G. max* parents were directed at selecting for *e1la*:K82E alleles and the desired *E1* alleles leading to the parent line KB16-2B #666, which was still segregating at *E1* and the parent line KB16-W2F3, which was fixed for *e1-as* (Table 3, Supplemental Figure 1, and Supplemental Figure 2). Desirable alleles of *tof11–1* and *tof12–*1 were also confirmed by genotyping for the parent lines (Supplemental Table 4). Additional breeding was conducted to reduce the *G. soja* genetic background for new parent lines with the *e1la*:K82E alleles, (Supplemental Fig. 1B and 1C). The resulting lines KB17–2#514 and KB17–1#481 were used both as new parent lines for populations 9 (*E1*) and 5, 6, and 7 (*e1-as*), respectively, as well as for phenotypic analysis as parts of the population 1 and 2 experimentally tested lines (Table 1; Table 2 and Supplemental Table 4).

A line carrying the *e1lb*:Del alleles (W82 FN) was identified from a set of fast neutron mutagenized Williams 82 lines, and lines with *E1* or *e1-as* alleles along with *e1lb*:Del alleles were selected by gentoype from two populations. Population 3 lines were selected for *e1lb*:Del/*E1* and population 4 lines were selected for *e1lb*:Del/*e1-as* and (Table 2).

Other populations were made following the general strategy breeding cycle of creating F_1_ seeds, advancing to F_2_ plants in winter nursery for genotypic selection of *e1la:K82E* with *E1*, *e1-as*, *e2*, or *e3;* harvest of F_2:3_ seeds; then one generation advance to F_3:4_ in Columbia, Missouri for seed supply for plots for the 2020 field experiment. The population information including the parents for each population is listed in Table 2, the parents and test lines are listed with their genotypes in Table 1, and the experimental categories and genotypes from the populations utilized for the field experiments are listed in Supplemental Table 4.

#### Evaluation of *e1la* and *e1lb* populations for flowering time and maturity

Two field experiments were conducted, one that included selections from populations 2–5 in 2018 and 2019 (18/19) and one that included selections from populations 6–10 in 2020 (20) (Table 2; Supplemental Table 4). For the 18/19 experiment, F_3_ lines fixed for *e1la*:K82E and *e1lb*:Del, along with *E1La* and *E1Lb* reference control lines, were planted in 3′ plots (1′ planted, 2′ alleys) on May 15th, 2018 at the South Farm Research Center in Columbia, Missouri. The following year, F_4_ lines fixed for *e1la*:K82E and *e1lb*:Del, along with *E1La* and *E1Lb* reference control lines, were planted in 5′ plots (3′ planted, 2′ alleys) on May 31st, 2019 in Columbia, Missouri. In both years, lines were grown in a randomized complete block design with three replicates per line and were scored for flowering time (R1) and maturity (R8) as a function of days after planting (DAP). In 2018, the R1 dates for each plant were averaged to get the mean R1 date for each plot. In 2019, plots were marked as R1 once flowers were observed on at least three plants in the plot. In both years, plots were marked as R8 once 95% of pods on the main stem were mature. First frost occurred on October 16th in 2018 (day 152) and on October 12th in 2019 (day 134). For the statistical analysis, any plot that did not mature by this time was given an R8 score of the day of the first frost. Means comparisons were conducted using an ANOVA in R, and significance groups were obtained using a Fisher’s LSD test with false discovery rate (FDR) correction (*P* = 0.05). For the 20 experiment, 50-seed plots of F_3:4_ lines along with controls were planted in random order in 5′ plots (3′ planted, 2′ alleys) on June 1st, 2020 in Columbia, Missouri. Genotypes were replicated, but lines were not. Plots were marked as R1 once flowers were observed on at least three plants in the plot, and maturity was estimated for 95% mature pods on the main stem, or maturity was forecasted 2–3 days in advance. The first frost occurred on October 16th in 2020 (day 137).

#### Geographic analysis of natural variation in *E1* and *E1La*

*E1* and *E1La* genotypes for 56 re-sequenced *Glycine soja* accessions were obtained from Zhou et al.*,* 2015 analyzed in SNPViz [24]. *E1* and *E1La* genotypes for 92 additional MG II or earlier *Glycine soja* accessions were obtained from Sanger sequencing. As North American cultivars derived from early maturity groups were underrepresented in our resequencing panel, cultivars with SoySNP50k data were instead pulled from the GRIN. The *E1* and *E1La* genotypes for each cultivar were estimated using a proxy SNP from the SoySNP50k array. *E1* and *E1La* genotypes were first determined for a set of 775 resequenced accessions [23, 25]. Strength of association between the *e1-as* and *e1la*:K82E causal mutations and all of the SoySNP50k variants within 1 Mbp of the causal mutations on chromosomes 06 and 04, respectively, was calculated using a parameter called“combined pessimistic accuracy.” Accuracy for each SNP is calculated as the percentage of the 775 resequenced accessions with either the REF or ALT haplotype combinations between SNP and causal mutation.

$$ Combined\ pessimistic\ accuracy=\left(\frac{\# of accessions\ with\ correct\  WT\  association+\#\# of\ accessions\ with\ correct\  MUT\  association}{Total\# of\ accessions}\right)\ x\ 100 $$

This “combined pessimistic accuracy” equation is modified from Metz (1978) to capture combined sensitivity, specificity, and missing data [37]. The single SNP on chromosomes 06 (ss715593865 – GM06:20916554; Accuracy = 94.1%) and 04 (ss715587601 – GM04:37750626; Accuracy = 92.0%) with the highest accuracy values were used to estimate *E1* and *E1La* genotypes for all North American cultivars pulled from the GRIN (only cultivars for which latitude and longitude coordinates could be obtained, and proxy SNP genotypes were homozygous, were used). To avoid overplotting on map figures, genotype groups containing more than 200 cultivars were randomly sampled. Latitude and longitude coordinates for plotting were obtained directly from the GRIN, where available. For accessions where coordinates were not available in GRIN, Google geocoding was used to obtain latitude and longitude coordinates for the state/province of origination. A small amount of “randomness” was introduced to plotting coordinates to prevent complete overlap of accessions originating from the same state/province. Maps were generated using ggplot2 in R using the Natural Earth package. Means comparisons for boxplots were conducted using an ANOVA in R, and significance groups were obtained using a Fisher’s LSD test with false discovery rate (FDR) correction (*P* = 0.05).

For the frequency distribution of imputed *e1la*:K82E alleles by country of origin, country of origin assignments for the 476 *G. max* and 499 *G. soja* accessions containing the *e1la*:K82E SoySNP50k Proxy SNP (ss715587601 – GM04:37750626) were obtained from the GRIN. Accessions missing country of origin information in the GRIN were assigned a value of “Unknown” for plotting. Histograms were generated in R v4.0.2 using the ggplot2 package, v3.3.2.

DNA was prepared as previously described from a single dry seed from 19 tofu and 26 natto experimental lines from the North Dakota State University soybean breeding program [14]. SimpleProbe genotyping assays for *E1*/*e1-as* and *E1La*/*e1la:K82E* were conducted as described above with the DNA samples and controls.

## Supplementary Information

**Additional file 1: Supplemental Figure 1.**Breeding schemes to transfer *e1la:K82E* alleles from *G. soja* PI 547831 into *G. max* background. A. Parents and experimental lines used to develop the experimental parent line KB16-2B #666. B. Lines used in development of the KB17–2 population. C. Lines used in the development of KB17–1 population. **Supplemental Figure 2.**Breeding scheme to transfer *e1la:K82E* alleles from *G. soja* PI 522226 into *G. max* background. **Supplemental Figure 3.**Frequency of GRIN-derived *Glycine max* (top panel) and *Glycine soja* (bottom panel) accessions containing the *e1la*:K82E proxy SNP by country of origin. Country of origin assignments for each accession were obtained from the GRIN. Accessions lacking country of origin information were assigned a value of “Unknown”. **Supplemental Table 1.** Flowering time and maturity genes affecting soybean photoperiod response. **Supplemental Table 2.** NCBI Blastp results for Legume orthologues of *E1La.* **Supplemental Table 3** List of 49 predicted genes deleted as a result of Fast Neutron-induced lesion. **Supplemental Table 4.** Maturity gene alleles for controls and test lines. **Supplemental Table 5.** Origin information for geographic assessment of *G. soja* accessions. **Supplemental Table 6.** Origin information for geographic assessment of North American cultivars. **Supplemental Table 7.** *E1* and *E1La* genotype status of North Dakota tofu breeding lines. **Supplemental Table 8.** *E1* and *E1La* genotype status of North Dakota natto breeding lines.

## Data Availability

The datasets supporting the conclusions of this article are included within the article (and its additional files). SoySNP50k genotype data for the *e1-as* and *e1la*:K82E proxy SNPs were obtained from Soybase (https://soybase.org/). Geographic coordinates and origin information for accessions were obtained from the Germplasm Resources Information Network search page (https://npgsweb.ars-grin.gov/gringlobal/search). The data from both repositories are freely accessible to the public for download. Seeds were obtained with permission from the USDA National Plant Germplasm System, from line or cultivar developers, or developed as part of this research.

## Abbreviations

CGH: Comparative genomic hybridization
CNV: Copy number variation
FDR: False discovery rate
FN: Fast neutrons
GRIN: Germplasm Resources Information Network
Mbp: Million base pairs
PI: Plant Introduction
REF: Reference

## References

[1] Carter T, Hymowitz T, Nelson R, Werner D (2004). Biological resources and migration.

[2] Bernard RL (1971). Two Major Genes for Time of Flowering and Maturity in Soybeans. Crop Science.

[3] Watanabe S, Xia Z, Hideshima R, Tsubokura Y, Sato S, Yamanaka N, Takahashi R, Anai T, Tabata S, Kitamura K, Harada K (2011). A map-based cloning strategy employing a residual heterozygous line reveals that the *GIGANTEA* gene is involved in soybean maturity and flowering. Genetics.

[4] Watanabe S, Hideshima R, Xia Z, Tsubokura Y, Sato S, Nakamoto Y, Yamanaka N, Takahashi R, Ishimoto M, Anai T, Tabata S, Harada K (2009). Map-based cloning of the gene associated with the soybean maturity locus *E3*. Genetics.

[5] Liu B, Kanazawa A, Matsumura H, Takahashi R, Harada K, Abe J (2008). Genetic redundancy in soybean Photoresponses associated with duplication of the *Phytochrome a* gene. Genetics.

[6] Bonato ER, Vello NA (1999). E6, a dominant gene conditioning early flowering and maturity in soybeans. Genet Mol Biol.

[7] Cober ER, Voldeng HD (2001). A new soybean maturity and photoperiod-sensitivity locus linked to *E1* and *T*. Crop Sci.

[8] Cober ER, Molnar SJ, Charette M, Voldeng HD (2010). A new locus for early maturity in soybean. Crop Sci.

[9] Kong F, Nan H, Cao D, Li Y, Wu F, Wang J, Lu S, Yuan X, Cober ER, Abe J, Liu B (2014). A new dominant gene *E9* conditions early flowering and maturity in soybean. Crop Sci.

[10] Samanfar B, Molnar SJ, Charette M, Schoenrock A, Dehne F, Golshani A, Belzile F, Cober ER (2017). Mapping and identification of a potential candidate gene for a novel maturity locus, *E10*, in soybean. Theor Appl Genet.

[11] Li M-W, Liu W, Lam H-M, Gendron JM (2019). Characterization of two growth period QTLs reveals modification of *PRR3* genes during soybean domestication. Plant Cell Physiol.

[12] Lu S, Dong L, Fang C, Liu S, Kong L, Cheng Q, Chen L, Su T, Nan H, Zhang D, Zhang L, Wang Z, Yang Y, Yu D, Liu X, Yang Q, Lin X, Tang Y, Zhao X, Yang X, Tian C, Xie Q, Li X, Yuan X, Tian Z, Liu B, Weller JL, Kong F (2020). Stepwise selection on homeologous *PRR* genes controlling flowering and maturity during soybean domestication. Nat Genet.

[13] Lu S, Zhao X, Hu Y, Liu S, Nan H, Li X, Fang C, Cao D, Shi X, Kong L, Su T, Zhang F, Li S, Wang Z, Yuan X, Cober ER, Weller JL, Liu B, Hou X, Tian Z, Kong F (2017). Natural variation at the soybean J locus improves adaptation to the tropics and enhances yield. Nat Genet.

[14] Langewisch T, Lenis J, Guo-Liang J, Wang D, Pantalone V, Bilyeu K (2017). The development and use of a molecular model for soybean maturity groups. BMC Plant Biol.

[15] Xia Z, Zhai H, Wu H, Xu K, Watanabe S, Harada K (2021). The synchronized efforts to decipher the molecular basis for soybean maturity loci E1, E2, and E3 that regulate flowering and maturity. Front Plant Sci.

[16] Xia Z, Watanabe S, Yamada T, Tsubokura Y, Nakashima H, Zhai H, Anai T, Sato S, Yamazaki T, Lü S (2012). Positional cloning and characterization reveal the molecular basis for soybean maturity locus E1 that regulates photoperiodic flowering. Proc Natl Acad Sci.

[17] Schmutz J, Cannon SB, Schlueter J, Ma J, Mitros T, Nelson W, Hyten DL, Song Q, Thelen JJ, Cheng J, Xu D, Hellsten U, May GD, Yu Y, Sakurai T, Umezawa T, Bhattacharyya MK, Sandhu D, Valliyodan B, Lindquist E, Peto M, Grant D, Shu S, Goodstein D, Barry K, Futrell-Griggs M, Abernathy B, du J, Tian Z, Zhu L, Gill N, Joshi T, Libault M, Sethuraman A, Zhang XC, Shinozaki K, Nguyen HT, Wing RA, Cregan P, Specht J, Grimwood J, Rokhsar D, Stacey G, Shoemaker RC, Jackson SA (2010). Genome sequence of the palaeopolyploid soybean. Nature.

[18] Xu M, Yamagishi N, Zhao C, Takeshima R, Kasai M, Watanabe S, Kanazawa A, Yoshikawa N, Liu B, Yamada T, Abe J (2015). The soybean-specific maturity gene *E1* family of floral repressors controls night-break responses through Down-regulation of *FLOWERING LOCUS T* Orthologs. Plant Physiol.

[19] Zhu J, Takeshima R, Harigai K, Xu M, Kong F, Liu B, Kanazawa A, Yamada T, Abe J (2019). Loss of function of the E1-like-b Gene Associates with early flowering under long-day conditions in soybean. Front Plant Sci.

[20] Xu M, Xu Z, Liu B, Kong F, Tsubokura Y, Watanabe S, Xia Z, Harada K, Kanazawa A, Yamada T, Abe J (2013). Genetic variation in four maturity genes affects photoperiod insensitivity and *PHYA*-regulated post-flowering responses of soybean. BMC Plant Biol.

[21] Takeshima R, Hayashi T, Zhu J, Zhao C, Xu M, Yamaguchi N, Sayama T, Ishimoto M, Kong L, Shi X, Liu B, Tian Z, Yamada T, Kong F, Abe J (2016). A soybean quantitative trait locus that promotes FLOWERING under long days is identified as *FT5a*, a *FLOWERING LOCUS T* ortholog. J Exp Bot.

[22] Liu L, Gao L, Zhang L, Cai Y, Song W, Chen L, Han T. Co-silencing E1 and its homologs in an extremely late-maturing soybean cultivar confers super-early maturity and adaptation to high-latitude short-season regions. Journal of Integrative Agriculture. 2020. 10.1016/S2095-3119(20)63391-3.

[23] Zhou Z, Jiang Y, Wang Z, Gou Z, Lyu J, Li W, Yu Y, Shu L, Zhao Y, Ma Y (2015). Resequencing 302 wild and cultivated accessions identifies genes related to domestication and improvement in soybean. Nat Biotechnol.

[24] Langewisch T, Zhang H, Vincent R, Joshi T, Xu D, Bilyeu K (2014). Major soybean maturity gene haplotypes revealed by SNPViz analysis of 72 sequenced soybean genomes. PLoS One.

[25] Valliyodan B, Brown AV, Wang J, Patil G, Liu Y, Otyama PI, Nelson RT, Vuong T, Song Q, Musket TA, Wagner R, Marri P, Reddy S, Sessions A, Wu X, Grant D, Bayer PE, Roorkiwal M, Varshney RK, Liu X, Edwards D, Xu D, Joshi T, Cannon SB, Nguyen HT (2021). Genetic variation among 481 diverse soybean accessions, inferred from genomic re-sequencing. Scientific Data.

[26] Stacey MG, Cahoon RE, Nguyen HT, Cui Y, Sato S, Nguyen CT, Phoka N, Clark KM, Liang Y, Forrester J, Batek J, Do PT, Sleper DA, Clemente TE, Cahoon EB, Stacey G (2016). Identification of Homogentisate dioxygenase as a target for vitamin E biofortification in oilseeds. Plant Physiol.

[27] Miranda C, Culp C, Škrabišová M, Joshi T, Belzile F, Grant DM, Bilyeu K (2019). Molecular tools for detecting Pdh1 can improve soybean breeding efficiency by reducing yield losses due to pod shatter. Mol Breed.

[28] Hosking R. A Dictionary of Japanese Food-Ingredients and Culture. North Claredon: Tuttle Publishing; 1995.

[29] Bolon Y-T, Haun WJ, Xu WW, Grant D, Stacey MG, Nelson RT, Gerhardt DJ, Jeddeloh JA, Stacey G, Muehlbauer GJ, Orf JH, Naeve SL, Stupar RM, Vance CP (2011). Phenotypic and genomic analyses of a fast neutron mutant population resource in soybean. Plant Physiol.

[30] Pham A-T, Lee J-D, Shannon JG, Bilyeu KD (2010). Mutant alleles of FAD2-1A and FAD2-1Bcombine to produce soybeans with the high oleic acid seed oil trait. BMC Plant Biol.

[31] Beuselinck PR, Sleper DA, Bilyeu KD (2006). An assessment of phenotype selection for linolenic acid using genetic markers. Crop Sci.

[32] Wang J, Chuang K, Ahluwalia M, Patel S, Umblas N, Mirel D, Higuchi R, Germer S (2005). High-throughput SNP genotyping by single-tube PCR with tm-shift primers. Biotechniques.

[33] Dierking EC, Bilyeu KD (2009). New sources of soybean seed meal and oil composition traits identified through TILLING. BMC Plant Biol.

[34] Shannon JG, Wrather JA, Sleper DA, Robbins RT, Nguyen HT, Anand SC (2007). Registration of ‘Jake’ soybean. J Plant Regist.

[35] Palacios MF, Easter RA, Soltwedel KT, Parsons CM, Douglas MW, Hymowitz T, Pettigrew JE (2004). Effect of soybean variety and processing on growth performance of young chicks and pigs1. J Anim Sci.

[36] Nelson RL, Johnson EOC (2012). Registration of the high-yielding soybean germplasm line LG04-6000. J Plant Regist.

[37] Metz CE (1978). Basic principles of ROC analysis. Semin Nucl Med.

