# Supplementary material for: Geographic distribution of the E1 family of genes and their effects on reproductive timing in soybean
Source: BMC Plant Biol. 2021 Sep 29;21:441. doi: 10.1186/s12870-021-03197-x (PMC8480027; doi:10.1186/s12870-021-03197-x)
Supplement: Supplementary file 1 — Additional file 1: Supplemental Figure 1.Breeding schemes to transfer e1la:K82E alleles from G. soja PI 547831 into G. max background. A. Parents and experimental lines used to develop the experimental parent line KB16-2B #666. B. Lines used in development of the KB17–2 population. C. Lines used in the development of KB17–1 population. Supplemental Figure 2.Breeding scheme to transfer e1la:K82E alleles from G. soja PI 522226 into G. max background. Supplemental Figure 3.Frequency of GRIN-derived Glycine max (top panel) and Glycine soja (bottom panel) accessions containing the e1la:K82E proxy SNP by country of origin. Country of origin assignments for each accession were obtained from the GRIN. Accessions lacking country of origin information were assigned a value of “Unknown”. Supplemental Table 1. Flowering time and maturity genes affecting soybean photoperiod response. Supplemental Table 2. NCBI Blastp results for Legume orthologues of E1La. Supplemental Table 3 List of 49 predicted genes deleted as a result of Fast Neutron-induced lesion. Supplemental Table 4. Maturity gene alleles for controls and test lines. Supplemental Table 5. Origin information for geographic assessment of G. soja accessions. Supplemental Table 6. Origin information for geographic assessment of North American cultivars. Supplemental Table 7. E1 and E1La genotype status of North Dakota tofu breeding lines. Supplemental Table 8. E1 and E1La genotype status of North Dakota natto breeding lines. [file 12870_2021_3197_MOESM1_ESM.pdf]

Supplemental Figure 1

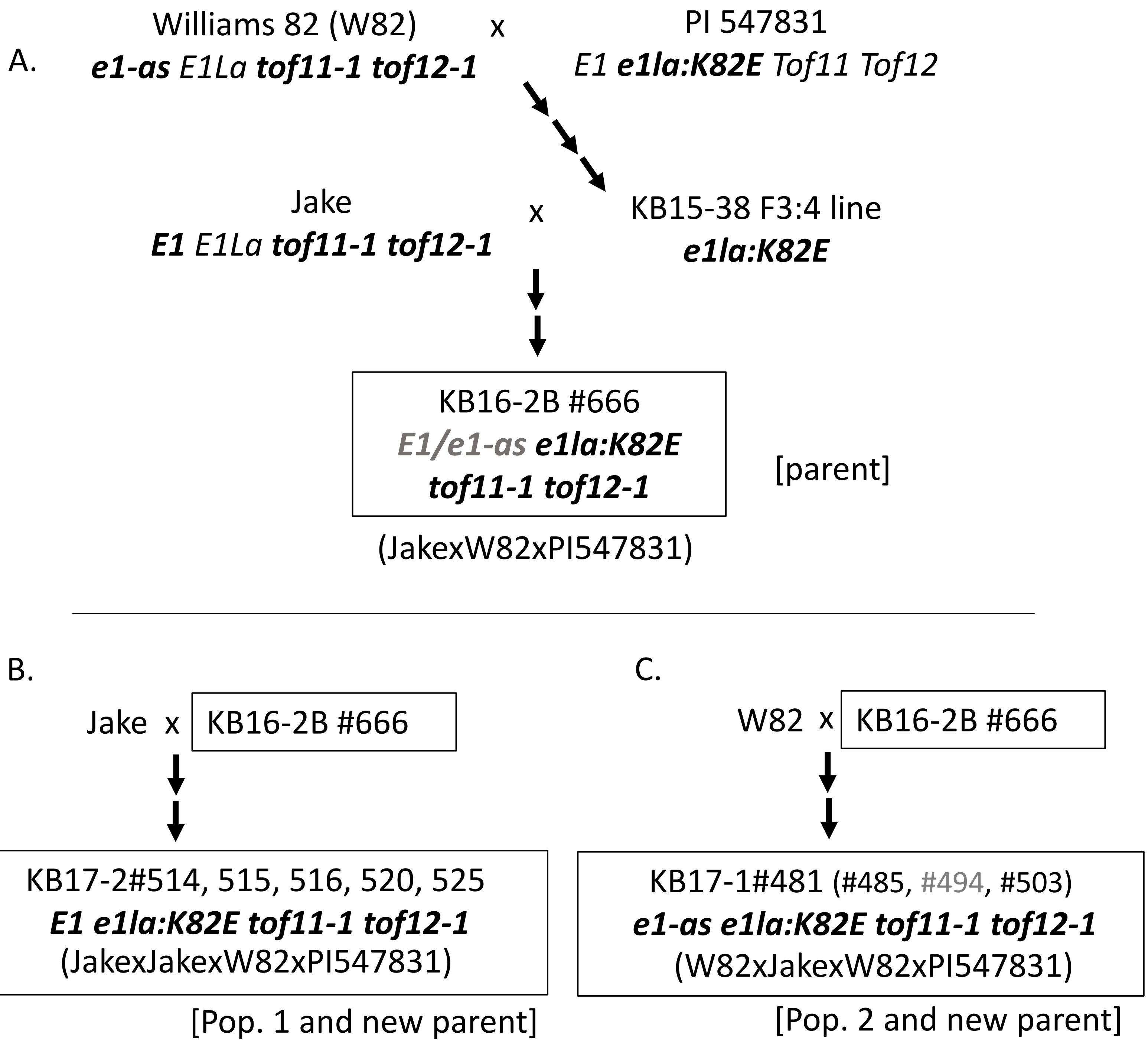

Supplemental Figure 1. Breeding schemes to transfer *e1la:K82E* alleles from *G. soja* PI 547831 into *G. max* background. A. Parents and experimental lines used to develop the experimental parent line KB16-2B #666. B. Lines used in development of the KB17-2 population. C. Lines used in the development of KB17-1 population.

Supplemental Figure 2

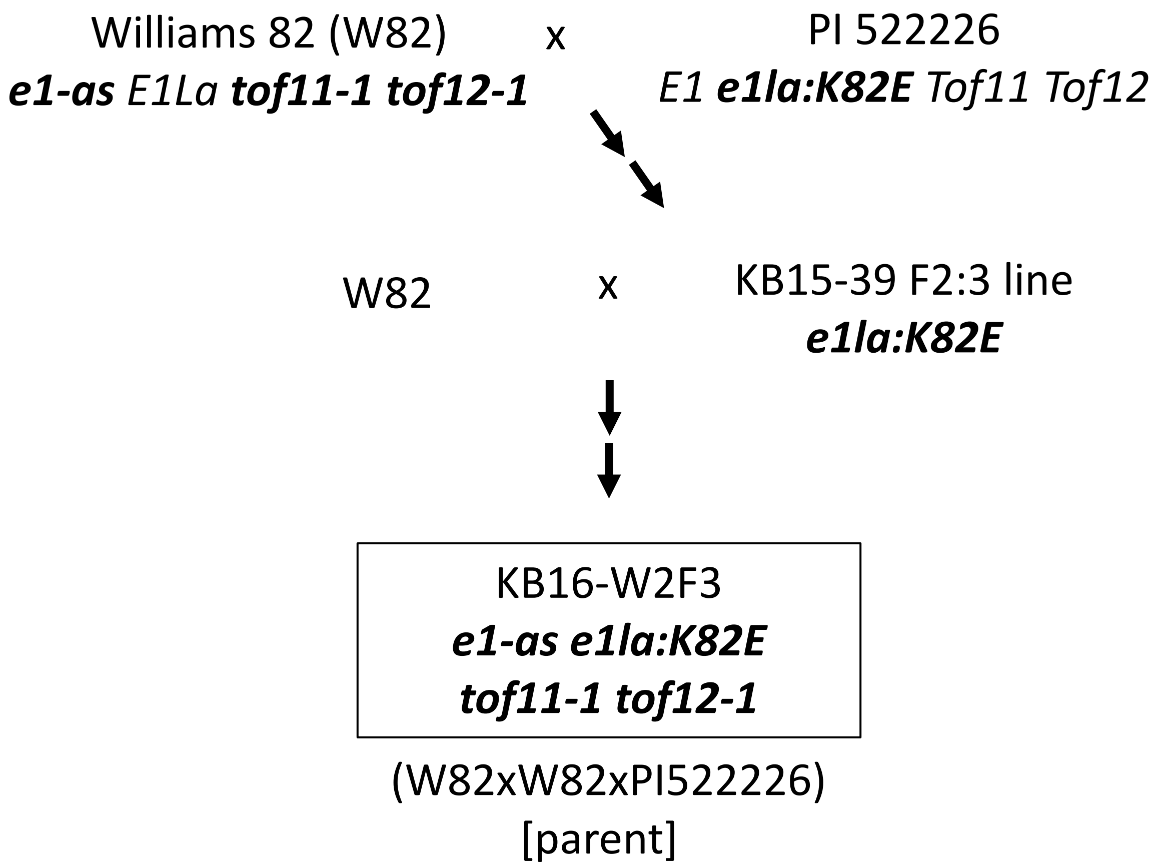

Supplemental Figure 2. Breeding scheme to transfer *e1la:K82E* alleles from *G. soja* PI 522226 into *G. max* background.

Supplemental Figure 3

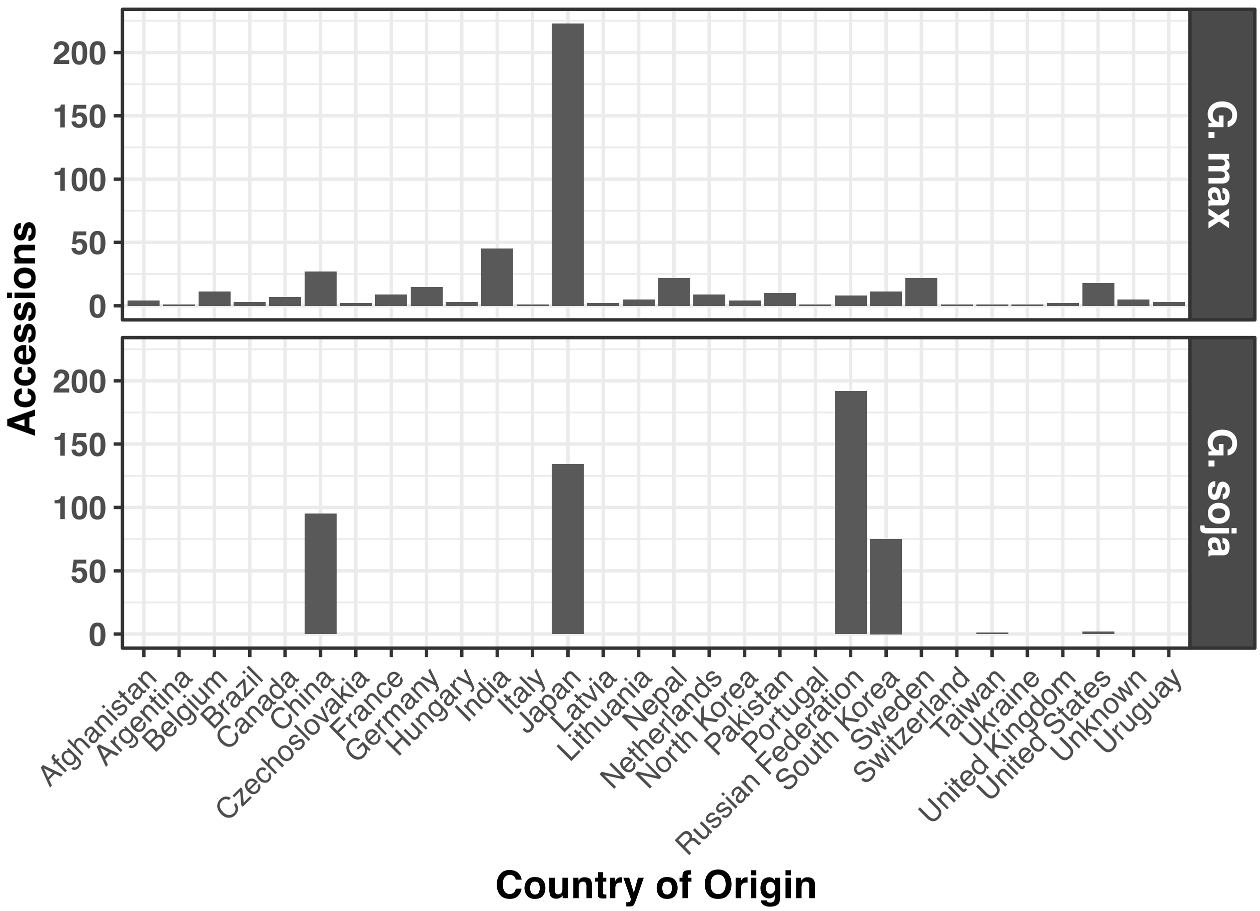

Supplemental Figure 3. Frequency of GRIN-derived *Glycine max* (top panel) and *Glycine soja* (bottom panel) accessions containing the *e1la*:K82E proxy SNP by country of origin. Country of origin assignments for each accession were obtained from the GRIN. Accessions lacking country of origin information were assigned a value of “Unknown.”

Supplemental Table 1. Flowering time and maturity genes affecting soybean photoperiod response

REF/ALT allele	Variant allele	Williams 82.a2.v1	Position (v2)	REF/ALT	v1	Position (v1)
e1-as/E1	T15R	Glyma.06g207800	20207322	C/G	Glyma06g23040	20007173
E1La/e1la	K82E	Glyma.04g156400	36758368	A/G	Glyma04g24640	28294378
E1Lb/e1lb	Deletion	Glyma.04G143300	26120011*	/deletion	Glyma18g22670	25739929
tof12-1/Tof12	stop627Q	Glyma.12g073900	5520945	C/T	Glyma12g07861	5510001
tof11-1/Tof11	frameshift	scaffold_32	198774	A/-	Glyma11g15580	11233895
E2/e2	K521stop	Glyma.10g221500	45310798	A/T	Glyma10g36600	44732850
E3/e3-tr	Deletion	Glyma.19g224200	47638567	/deletion	Glyma19g41210	47516604

*The start codon position is indicated, but the entire gene sequence is deleted in *e1lb* :Del.

Supplemental Table 2. NCBI Blastp results for Legume orthologues of *E1La*

>Glycine max
MSNPSDEKEQCQKKRKSTICEASNFKTSRRRFFSNKNEEDMNKGVSTTLKLYDDPWKIKKTLTDSDLGILSRLSLAADLVKKQILPMLGADHARAAETEEGTPVRVWDMDTKSMHQLVLKRWSSSKSYVLIGKWNQDFVRRRDLKKGDEIGFHWDPYNCVFNFCVLKRAMPEN
>Glycine soja
MSNPSDEKEQCQKKRKSTICEASNFKTSRRRFFSNKNEEDMNKGVSTTLKLYDDPWKIKKTLTDSDLGILSRLSLAADLVKKQILPMLGADHARAAETEEGTPVRVWDMDTKSMHQLVLKRWSSSKSYVLIGKWNQDFVRRRDLKKGDEIGFHWDPYNCVFNFCVLKRAMPEN
>Arachis duranensis
VSTTLKLYDDPWKIKKTLTDSDLGILSRLLLAADLVKKQILPMLGGDHARAAETEEGTPVRVWDMDTRSMHHLVLKRWSSSKSYVLIGKWNQDFVRRRDLKKGDEIGFHWDPYNCAFNFCVLTRA
>Arachis hypogaea
VSTTLKLYDDPWKIKKTLTDSDLGILSRLLLAADLVKKQILPMLGGDHARAAETEEGTPVRVWDMDTRSMHHLVLKRWSSSKSYVLIGKWNQDFVRRRDLKKGDEIGFHWDPYNCAFNFCVLTRA
>Arachis ipaensis
VSTTLKLYDDPWKIKKTLTDSDLGILSRLLLAADLVKKQILPMLGGDDARAAETEEGTPVRVWDMDTRSMHHLVLKRWSSSKSYVLIGKWNQDFVRRRDLKKGDEIGFHWDPYNCAFNFCVLTRA
>Phaseolus vulgaris
MSNPGDEKELCQKKRKSTICEASNFRTSRRRFCSNQSEEEMNKGVSTTLKLYDDPWKIKKTLTDSDLGILSRLLLAADLVKKQILPMLGADHARAAETEEGTPVRVWDIDTKSMHQLVLKRWSSSKSYVLIGKWNQDFVRRRDLKKGDEIGFHWDPYNCIFNFCVLKRAMPEN
>Vigna angularis
MSNPGDEKELCQKKRKSTICEASNFRTSRRRFCSSNQIEEEMNKGVSTTLKLYDDPWKIKKTLTDSDLGILSRLLLAADLVKKQILPMLGADHARAAETEEGTPVRVWDMDTKSMHQLVLKRWSSSKSYVLIGKWNQDFVRRRDLKKGDEIGFHWDPYNCIFNFCVLKRAMPDN
>Mucuna pruriens
MNNPTDEREQCQKKRKSTICEASTFRSSRRRFCNNNNEEEMNKGVSTTLKLYDDPWKIKKTLTDSDLGILSRLLLAADLVKKQILPMLGADHARAAETEEGTPVRVWDMDTKSMHQLVLKRWSSSKSYVFIGKWNQDFVRRRDLKKGDEIGFHWDPYNCVFNFCVLKRAMLEN
>Abrus precatorius
MNNPIDEREQCQKKRKSTICEASTLRTSRRRFYNNKNEEERNKGVSTTLKLYDDPWKIKKTLTDSDLGILSRLLLAADLVKKQILPMLSVDHARAAETEEGTPVKVWDMDTKSMHQLVLKRWSSSKSYVLIGKWNQDFVRRRDLKKGDEIGFHWDPYNCAFNFCVLKRAVPEN
>Vigna radiata var. radiata
MSNPGDEKELCQRKRKSTICEASNFRTSRRRFCSNQIEE----GVSTTLKLYDDPWKIKKTLTDSDLGILSRLLFAADLVKKQILPMLGADHARAAETEEGTPVRVWDMDTKSMHQLVLKRWSSSKSYVLIGKWNQDFVRRRDLKKGDEIGFHWDPYNCIFNFCVLKRAIPDN
>Spatholobus suberectus
MNNPADEREQCQKKRKSITCEASTLRTSRRRFCNNKNEEEMNKGVSTTLKLYDDPWKIKKKLTDSDLGILSRLLLAADLVKKQILPLLGADHARAAETEEGTPVRIWDMDTKTMHQLVLKRWSSSKSYVLIGKWNQDFVRRRDLKKGDEIGFHWDPYNCVFNFCVLKRTMPEN
>Cajanus cajan
MSNPHADEREKCQKKRKSTICESSSLRTSRRRYCNNKNEEEMNKEVSTTLKLYDDPWKIKKTLTDSDLGILSRLLLATDLVKKQILPMLGVDHARAAEIEEGTPISVWDIDTKSMHQLILKQWSSSKSYVLIGKWNQDFVRRRDLKKGDEIGFHWDPYNCVFNFCVLKRVMSEN
>Prosopis alba
VSTALKLYEDPWKIKKTLTESDLGILSRLLLAADMVKRHILPMLGGEGARAAESEEGTQVRVWDTDTKSMHQLLLKRWSSSKSYVLIGKWSQDFVRRRELKKGDEIGFYWDPYTSLFNFCVLKRA
>Cicer arietinum
GDEKEQCQKKRKSCDETSTNIYMRTSRKRLCSNKNEEDRNNNDNNKGSVSTTLKLYDDPWKIKKTLTDSDLGILSRLLLAADLVKKQILPMLDVDDARAAETEEGSPVNVWDMETKSMHQLVLKRWSSSKSYVLIGKWNQDFVRRRELKKGDEIGFQWDPFNRAFNFCVLKRTM
>Trifolium medium
GEEREQYQKKRKSCDEATTNLRTSRRRLCNNKNEEDGHNYNKSVSTTLKLYDDPWKIKKTLTDSDLGILSRLLLAADLVKKQILPMLDLDNARAAETEEGSPVNVWDMETNSMHQLVLKRWSSSKSYVLIGKWNHDFVRRRDLKKGDEIGFQWDPFNRAFNFCVLKRA
>Trifolium pratense
GEEREQYPKKRKSCDEATTNMRTSRRRLCNNKNEEDGHNYNKSVSTTLKLYDDPWKIKKTLTDSDLGILSRLLLAADLVKKQILPMLDLDDARAAETEEGSPVNVWDMETNSMHQLVLKRWSSSKSYVLIGKWNHDFVRRRDLKKGDEIGFQWDPFNRAFNFCVLKRA
>Lotus japonicus
MNNLGDETEFCQRKRKSPSSEGS---TSRRRFSSNNNNE--KDGVSTTLKLYDDPWKIKKTLMASDLGILSRLMLAADLVKKQILPMLGVHQARAAETEQGSQVRVWDVDTESMHQLVLKRWSSSKSYVLIGKWSQDFVRRRELKKGDEIGFYWDPYNCAFNFCVLKRA
>Medicago truncatula
EMEQLQKKRKSCDEASTNLKTSRRRLCNNKNEEQNNNQNNDNKGSVSTTLKLYDDPWKIKKSLTESDLGILSRLLLAADLVKKQILPMLDVDDARAAETEEGSPVNVWDMETNSMHELVLKRWSSSKSYVLIGKWNQDFVRRRELKKGDEIGFQWDPFNRAFNFCVLKRAIP
>Senna tora
TALKLYEDPWTIKKTLTESDLGRSSRLLLATDVVKSEILPMLGVDDARAAESEEGAAVRVWDLDSKSMHHLLLKRWSSSKSYVFIGKWNHDFVRRRNLHKGDLIAFHWDPSHSLFNFC
>Trifolium subterraneum
GEEREQYQKKRKSCDEASTNLRTSKRRLCNNKNEEEGNNYNKSISTTLKLYDDPWKIKKTLTDSDLGILSRLLLAADLVKKQILPMLDLDDARAAETEEGSPVNVWDVETNSMHQLVLKRWSSSKSYVLIGKWNHDFVRRRDLKKGDEIGFQWDPFNRAFNFCVLKRATMPQ
>Lupinus albus
NKESQYWGCSTSLELYDNPWKIKKVLTTSDLGKLNRLLFGADLLENLMLPVLGASAQRDAESGMGTPIRVWDVDTMSMHMLILKRWASFKNYVLIGKWNHEFVRRRELKKGDEIGLQWDSYRHCFNFSVLKR
>Lupinus angustifolius
NKESQYWGCSTSLELYDNPWKIKKVLTTSDLGKLNRLLFGAELLENLMLPVLGVAAQRDAESGMGTPIRVWDVDTMSMHMLILKRWASFKNYVLIGKWNHEFVRRRELKKGDEIGLQWDSYRHCFNFSVLKRT
>Vigna unguiculata
STSLELYENPWKIRKVLTKSDTGRLSRLLLGADVGENFMLPVLDSHAQTEVINGTGSTVSVWDVDTMSMHNLILKRWPSFNNFVLMGRWSSDFVQRRQLIKGDEIGLLWDSFKHCFHFSVLKR

Supplemental Table 3 List of 49 predicted genes deleted as a result of Fast Neutron-induced lesion.

G. max (v1)	G. max (v2)	Chr	Gene Coordinates	Annotation	Arabidopsis Orthologue
Glyma18g22170	Glyma.04G142900.1	Gm18	24890295..24893116	ATP citrate lyase (ACL) family protein	AT2G20420.1
Glyma18g22256	Glyma.04G143000.1	Gm18	25169952..25194865	diacylglycerol kinase 7	AT4G30340.1
Glyma18g22256	Glyma.04G143000.2	Gm18	25169952..25194865	diacylglycerol kinase 4	AT5G57690.1
Glyma18g22256	Glyma.04G143000.3	Gm18	25169952..25194865	diacylglycerol kinase 4	AT5G57690.1
Glyma18g22256	Glyma.04G143000.4	Gm18	25169952..25194865	diacylglycerol kinase 7	AT4G30340.1
Glyma18g22256	Glyma.04G143000.5	Gm18	25169952..25194865	diacylglycerol kinase 3	AT2G18730.1
Glyma18g22256	Glyma.04G143000.6	Gm18	25169952..25194865	diacylglycerol kinase 3	AT2G18730.1
Glyma18g22256	Glyma.04G143000.7	Gm18	25169952..25194865	diacylglycerol kinase 3	AT2G18730.1
Glyma18g22256	Glyma.04G143000.8	Gm18	25169952..25194865	diacylglycerol kinase 3	AT2G18730.1
Glyma18g22343	Glyma.04G143100.1	Gm18	25307210..25309307	RNA-binding (RRM/RBD/RNP motifs) family protein	AT1G22910.3
Glyma18g22430	Glyma.04G143200.1	Gm18	25313564..25315368	Pectin lyase-like superfamily protein	AT3G07840.1
Glyma18g22670*	Glyma.04G143300.1	Gm18	25739929..25740831	AP2/B3-like transcriptional factor family protein (E1Lb)	AT2G33720.1
Glyma18g22696	Glyma.04G143400.1	Gm18	25749079..25752312	Cytidine/deoxycytidylate deaminase family protein	AT5G28050.2
Glyma18g22723	Glyma.04G143800.1	Gm18	25860331..25861059	RING/U-box superfamily protein	AT3G20395.1
Glyma18g22760	Glyma.04G143900.1	Gm18	25880524..25882018	no functional annotations for this locus	AT5G61820.1
Glyma18g22770	Glyma.04G144000.1	Gm18	25892137..25893631	no functional annotations for this locus	AT5G61820.1
Glyma18g22780	Glyma.04G144300.1	Gm18	25930817..25935749	enolase 1	AT1G74030.1
Glyma18g22790	Glyma.04G144500.1	Gm18	25965023..25967991	tetraspanin11	AT1G18520.1
Glyma18g22800	Glyma.04G144600.1	Gm18	25972253..25983796	acyl-activating enzyme 17	AT5G23050.1
Glyma18g22811	Glyma.04G144700.1	Gm18	26012981..26013430	Far-red impaired responsive (FAR1) family protein	AT3G59470.1
Glyma18g22870	Glyma.04G144800.1	Gm18	26107214..26107696	EF hand calcium-binding protein family	AT1G18530.1
Glyma18g22880	Glyma.04G144900.1	Gm18	26108152..26114981	ATPase E1-E2 type family protein / haloacid dehalogenase-like hydrolase family prot	AT1G68710.1
Glyma18g22896	Glyma.04G145000.1	Gm18	26148576..26150496	nuclear factor Y, subunit B13	AT5G23090.1
Glyma18g22930	Glyma.04G145200.1	Gm18	26224576..26229437	ATP binding microtubule motor family protein	AT1G18550.1
Glyma18g22935	Glyma.04G145300.1	Gm18	26288428..26288849	white-brown complex homolog protein 11	AT1G17840.1
Glyma18g22940	Glyma.04G145400.1	Gm18	26290937..26296982	P-loop containing nucleoside triphosphate hydrolases superfamily protein	AT3G18600.1
Glyma18g22960	Glyma.04G145600.1	Gm18	26371172..26373671	Adenine nucleotide alpha hydrolases-like superfamily protein	AT1G48960.1
Glyma18g23000	Glyma.04G145700.1	Gm18	26509040..26510053	no functional annotations for this locus	AT1G49000.1
Glyma18g23102	Glyma.04G145800.1	Gm18	26644531..26646436	seed imbibition 2	AT3G57520.1
Glyma18g23174	Glyma.04G145900.1	Gm18	26706946..26707527	Plant mitochondrial ATPase, F0 complex, subunit 8 protein	AT2G07707.1
Glyma18g23247	Glyma.04G146000.1	Gm18	26952217..26971897	Transcription factor TFIIE, alpha subunit	AT1G03280.1
Glyma18g23397	Glyma.04G146200.1	Gm18	27099270..27099755	Protein phosphatase 2C family protein	AT2G33700.1
Glyma18g23454	Glyma.04G146300.1	Gm18	27153699..27158258	dsRNA-binding domain-like superfamily protein	AT1G09700.1
Glyma18g23580	Glyma.04G146500.1	Gm18	27163937..27164434	homolog of histone chaperone HIRA	AT3G44530.1
Glyma18g23590	Glyma.04G146600.1	Gm18	27166340..27178954	Nucleic acid-binding, OB-fold-like protein	AT5G63690.1
Glyma18g22750	Now new ID found	Gm18	25870288..25871782	no functional annotations for this locus	AT5G61820.1
Glyma18g22775	Now new ID found	Gm18	25929539..25930177	no functional annotations for this locus	
Glyma18g22820	Now new ID found	Gm18	26022017..26024355	aldehyde dehydrogenase 3I1	AT4G34240.1
Glyma18g22830	Now new ID found	Gm18	26033264..26034059	translation initiation factor 3 subunit H1	AT1G10840.1
Glyma18g22860	Now new ID found	Gm18	26046032..26048016	NAP1-related protein 2	AT1G18800.1
Glyma18g22913	Now new ID found	Gm18	26162392..26164515	Protein of unknown function, DUF617	AT5G23100.1
Glyma18g22973	Now new ID found	Gm18	26378446..26380409	BED zinc finger ;hAT family dimerisation domain	AT3G42170.1
Glyma18g22986	Now new ID found	Gm18	26495460..26499248	no functional annotations for this locus	
Glyma18g23030	Now new ID found	Gm18	26542611..26546777	extra-large GTP-binding protein 3	AT1G31930.1
Glyma18g23320	Now new ID found	Gm18	26973925..26976757	Hypoxia-responsive family protein	AT3G05550.1
Glyma18g23340	Now new ID found	Gm18	26982652..26984276	Insulinase (Peptidase family M16) protein	AT5G56730.1
Glyma18g23512	Now new ID found	Gm18	27157975..27159453	cytidine deaminase 1	AT2G19570.1
Glyma18g23570	Now new ID found	Gm18	27161786..27162426	no functional annotations for this locus	AT5G53650.1
Glyma18g23750	Now new ID found	Gm18	27180132..27183194	BED zinc finger ;hAT family dimerisation domain	AT3G42170.1

*Indicates the G. max (v1) glyma model corresponding to the *E1Lb* gene on Chr18 (moved to Chr04 in G. max [v2])

Supplemental Table 4. Maturity gene alleles for controls and test lines

ID	Name	*E1*	*E1La*	*E1Lb*	*E2*	*E3*	Tof11-1	Tof12-1	# of Plots (18/19/20)
18/19: e1_e1la	Pop 2	*e1-as*	*e1la* :K82E	REF	REF	REF	REF	REF	9/14/0
18/19: e1_e1lb	Pop. 4; W82 FN	*e1-as*	REF	*e1lb* :Del	REF	REF	REF	REF	39/23/0
18/19: E1_e1la	Pop. 1	*E1*	*e1la* :K82E	REF	REF	REF	REF	REF	12/11/0
18/19: E1_e1lb	Pop. 3	*E1*	REF	*e1lb* :Del	REF	REF	REF	REF	3/15/0
18/19: e1_E1La_E1Lb	W82, LG04-6000	*e1-as*	REF	REF	REF	REF	REF	REF	12/6/x
18/19: E1_E1La_E1Lb	Jake	*E1*	REF	REF	REF	REF	REF	REF	6/3/x
20: e1_e1la	Pop. 7; Pop. 8	*e1-as*	*e1la* :K82E	REF	REF	REF	REF	REF	0/0/10
20: e2_e1la	Pop. 5	*e1-as*	*e1la* :K82E	REF	*e2*	REF	REF	REF	0/0/6
20: e3_e1la	Pop. 6	*e1-as*	*e1la* :K82E	REF	REF	*e3*	REF	REF	0/0/3
20: E1_e1la	Pop. 9	*E1*	*e1la* :K82E	REF	REF	REF	REF	REF	0/0/2
20: e2_E1La_E1Lb	Deuel, Candor	*e1-as*	REF	REF	*e2*	REF	REF	REF	0/0/2
20: e3_E1La_E1Lb	Brookings	*e1-as*	REF	REF	REF	*e3*	REF	REF	0/0/1
20: e1_E1La_E1Lb	W82, LG04-6000	*e1-as*	REF	REF	REF	REF	REF	REF	0/0/2
20: E1_E1La_E1Lb	Jake, Ellis HOLL	*E1*	REF	REF	REF	REF	REF	REF	0/0/2

Supplemental Table 5. Origin information for geographic assessment of *G. soja* accessions

Source	Name	Origin	*E1*	*E1La*	Latitude*	Longitude*
GRIN	PI 423989A	Amur, Russian Federation	*E1*	*e1la:K82E*	52.9775503	127.362087
GRIN	PI 423992	Amur, Russian Federation	*E1*	*e1la:K82E*	52.9775503	127.362087
GRIN	PI 423996	Amur, Russian Federation	*E1*	*e1la:K82E*	52.9775503	127.362087
GRIN	PI 423997	Amur, Russian Federation	*E1*	*e1la:K82E*	52.9775503	127.362087
GRIN	PI 424000	Amur, Russian Federation	*E1*	*e1la:K82E*	52.9775503	127.362087
GRIN	PI 507719	Amur, Russian Federation	*E1*	*e1la:K82E*	54.6035065	127.480172
GRIN	PI 507722	Amur, Russian Federation	*E1*	*e1la:K82E*	52.9775503	127.362087
GRIN	PI 507723A	Amur, Russian Federation	*E1*	*e1la:K82E*	54.6035065	127.480172
GRIN	PI 507724	Amur, Russian Federation	*E1*	*e1la:K82E*	54.6035065	127.480172
GRIN	PI 507725A	Amur, Russian Federation	*E1*	*e1la:K82E*	54.6035065	127.480172
GRIN	PI 507725B	Amur, Russian Federation	*E1*	*e1la:K82E*	54.6035065	127.480172
GRIN	PI 507727	Amur, Russian Federation	*E1*	*e1la:K82E*	54.6035065	127.480172
GRIN	PI 507733	Amur, Russian Federation	*E1*	*e1la:K82E*	54.6035065	127.480172
GRIN	PI 507734	Amur, Russian Federation	*E1*	*e1la:K82E*	54.6035065	127.480172
GRIN	PI 507736	Amur, Russian Federation	*E1*	*e1la:K82E*	54.6035065	127.480172
GRIN	PI 507737	Amur, Russian Federation	*E1*	*e1la:K82E*	54.6035065	127.480172
GRIN	PI 507738	Amur, Russian Federation	*E1*	*e1la:K82E*	54.6035065	127.480172
GRIN	PI 507739A	Amur, Russian Federation	*E1*	*e1la:K82E*	54.6035065	127.480172
GRIN	PI 507739B	Amur, Russian Federation	*E1*	*e1la:K82E*	54.6035065	127.480172
GRIN	PI 507740	Amur, Russian Federation	*E1*	*e1la:K82E*	54.6035065	127.480172
GRIN	PI 507741A	Amur, Russian Federation	*E1*	*e1la:K82E*	54.6035065	127.480172
GRIN	PI 507742	Amur, Russian Federation	*E1*	*e1la:K82E*	54.6035065	127.480172
GRIN	PI 507743	Amur, Russian Federation	*E1*	*e1la:K82E*	54.6035065	127.480172
GRIN	PI 507746	Amur, Russian Federation	*E1*	*e1la:K82E*	54.6035065	127.480172
GRIN	PI 507747	Amur, Russian Federation	*E1*	*e1la:K82E*	54.6035065	127.480172
GRIN	PI 507748	Amur, Russian Federation	*E1*	*e1la:K82E*	54.6035065	127.480172
GRIN	PI 507749	Amur, Russian Federation	*E1*	*e1la:K82E*	54.6035065	127.480172
GRIN	PI 507750	Amur, Russian Federation	*E1*	*e1la:K82E*	54.6035065	127.480172
GRIN	PI 507753	Amur, Russian Federation	*E1*	*e1la:K82E*	54.6035065	127.480172
GRIN	PI 507754	Amur, Russian Federation	*E1*	*e1la:K82E*	54.6035065	127.480172
GRIN	PI 507758	Amur, Russian Federation	*E1*	*e1la:K82E*	54.6035065	127.480172
GRIN	PI 507759	Amur, Russian Federation	*E1*	*e1la:K82E*	54.6035065	127.480172
GRIN	PI 507760	Amur, Russian Federation	*E1*	*e1la:K82E*	54.6035065	127.480172
GRIN	PI 507762	Amur, Russian Federation	*E1*	*e1la:K82E*	54.6035065	127.480172
GRIN	PI 507763	Amur, Russian Federation	*E1*	*e1la:K82E*	54.6035065	127.480172
GRIN	PI 507764	Amur, Russian Federation	*E1*	*e1la:K82E*	54.6035065	127.480172
GRIN	PI 507811	Amur, Russian Federation	*E1*	*e1la:K82E*	54.6035065	127.480172
GRIN	PI 507813	Amur, Russian Federation	*E1*	*e1la:K82E*	54.6035065	127.480172
GRIN	PI 507814	Amur, Russian Federation	*E1*	*e1la:K82E*	54.6035065	127.480172
GRIN	PI 507818B	Amur, Russian Federation	*E1*	*e1la:K82E*	54.6035065	127.480172
GRIN	PI 507820	Amur, Russian Federation	*E1*	*e1la:K82E*	54.6035065	127.480172
GRIN	PI 507822	Amur, Russian Federation	*E1*	*e1la:K82E*	54.6035065	127.480172
GRIN	PI 507824	Amur, Russian Federation	*E1*	*e1la:K82E*	54.6035065	127.480172
GRIN	PI 507825	Amur, Russian Federation	*E1*	*e1la:K82E*	54.6035065	127.480172
GRIN	PI 507833	Amur, Russian Federation	*E1*	*e1la:K82E*	54.6035065	127.480172
GRIN	PI 507836	Amur, Russian Federation	*E1*	*e1la:K82E*	54.6035065	127.480172
GRIN	PI 507837	Amur, Russian Federation	*E1*	*e1la:K82E*	54.6035065	127.480172
GRIN	PI 507838B	Amur, Russian Federation	*E1*	*e1la:K82E*	54.6035065	127.480172
GRIN	PI 507839	Amur, Russian Federation	*E1*	*e1la:K82E*	54.6035065	127.480172
GRIN	PI 507844B	Amur, Russian Federation	*E1*	*e1la:K82E*	54.6035065	127.480172
GRIN	PI 507845	Amur, Russian Federation	*E1*	*e1la:K82E*	54.6035065	127.480172
GRIN	PI 522193	Amur, Russian Federation	*E1*	*e1la:K82E*	52.9775503	127.362087
GRIN	PI 538411A	Amur, Russian Federation	*E1*	*e1la:K82E*	52.9775503	127.362087
GRIN	PI 538411B	Amur, Russian Federation	*E1*	*e1la:K82E*	52.9775503	127.362087
GRIN	PI 567196	Amur, Russian Federation	*E1*	*e1la:K82E*	54.6035065	127.480172
GRIN	PI 578336	Amur, Russian Federation	*E1*	*e1la:K82E*	52.9775503	127.362087
GRIN	PI 639619	Amur, Russian Federation	*E1*	*e1la:K82E*	54.6035065	127.480172
GRIN	PI 458535	Heilongjiang, China	*E1*	*e1la:K82E*	48.2666683	126.600002
GRIN	PI 464866A	Heilongjiang, China	*E1*	*e1la:K82E*	48.1082859	127.074932
GRIN	PI 522179	Heilongjiang, China	*E1*	*e1la:K82E*	50.2128163	126.81559
GRIN	PI 522180	Heilongjiang, China	*E1*	*e1la:K82E*	48.8191949	128.407521
GRIN	PI 522181	Heilongjiang, China	*E1*	*e1la:K82E*	48.2666683	126.600002
GRIN	PI 522184	Heilongjiang, China	*E1*	*e1la:K82E*	45.7333374	127.450001
GRIN	PI 597450B	Heilongjiang, China	*E1*	*e1la:K82E*	46.8360643	130.359907
GRIN	PI 578342B	Khabarovsk, Russian Federatio	*E1*	*e1la:K82E*	48.4969043	135.132317
GRIN	PI 464926	Liaoning, China	*E1*	*e1la:K82E*	42.7228358	124.331345
GRIN	PI 464927C	Liaoning, China	*E1*	*e1la:K82E*	42.4642353	124.040257
GRIN	PI 507782	Primorye, Russian Federation	*E1*	*e1la:K82E*	45.0525641	135
GRIN	PI 507800A	Primorye, Russian Federation	*E1*	*e1la:K82E*	45.0525641	135
GRIN	PI 507800B	Primorye, Russian Federation	*E1*	*e1la:K82E*	45.0525641	135
GRIN	PI 507803	Primorye, Russian Federation	*E1*	*e1la:K82E*	45.0525641	135
GRIN	PI 522203	Primorye, Russian Federation	*E1*	*e1la:K82E*	44.9999962	134.999992
GRIN	PI 522206	Primorye, Russian Federation	*E1*	*e1la:K82E*	44.9999962	134.999992
GRIN	PI 578338A	Primorye, Russian Federation	*E1*	*e1la:K82E*	44.9999962	134.999992
GRIN	PI 507729	Amur, Russian Federation	*e1-as*	*E1La*	54.6035065	127.480172
GRIN	PI 507751	Amur, Russian Federation	*e1-as*	*E1La*	54.6035065	127.480172
GRIN	PI 507752	Amur, Russian Federation	*e1-as*	*E1La*	54.6035065	127.480172
GRIN	PI 507767	Amur, Russian Federation	*e1-as*	*E1La*	64.4131644	144.034228
GRIN	PI 507812A	Amur, Russian Federation	*e1-as*	*E1La*	54.6035065	127.480172
GRIN	PI 507826	Amur, Russian Federation	*e1-as*	*E1La*	54.6035065	127.480172
GRIN	PI 507827	Amur, Russian Federation	*e1-as*	*E1La*	54.6035065	127.480172
GRIN	PI 507828	Amur, Russian Federation	*e1-as*	*E1La*	54.6035065	127.480172

GRIN	PI 507830B	Amur, Russian Federation	*e1-as*	*E1La*	54.6035065	127.480172
GRIN	PI 507831	Amur, Russian Federation	*e1-as*	*E1La*	54.6035065	127.480172
GRIN	PI 507832	Amur, Russian Federation	*e1-as*	*E1La*	54.6035065	127.480172
GRIN	PI 507834	Amur, Russian Federation	*e1-as*	*E1La*	54.6035065	127.480172
GRIN	PI 507835	Amur, Russian Federation	*e1-as*	*E1La*	54.6035065	127.480172
GRIN	PI 507838A	Amur, Russian Federation	*e1-as*	*E1La*	54.6035065	127.480172
GRIN	PI 507840	Amur, Russian Federation	*e1-as*	*E1La*	54.6035065	127.480172
GRIN	PI 507841A	Amur, Russian Federation	*e1-as*	*E1La*	54.6035065	127.480172
GRIN	PI 507844A	Amur, Russian Federation	*e1-as*	*E1La*	54.6035065	127.480172
GRIN	PI 567194	Amur, Russian Federation	*e1-as*	*E1La*	52.9775503	127.362087
Zhou et al. 2015	PI 458538	Heilongjiang, China	*E1*	*e1la*	47.1216472	128.738231
Zhou et al. 2015	PI 547831	Illinois, United States	*E1*	*e1la*	40.6331249	-89.3985283
Zhou et al. 2015	PI 407288	Jilin, China	*E1*	*e1la*	43.837883	126.549572
Zhou et al. 2015	PI 447004	Jilin, China	*E1*	*e1la*	43.837883	126.549572
Zhou et al. 2015	PI 479752	Jilin, China	*E1*	*e1la*	43.837883	126.549572
Zhou et al. 2015	PI 407197	Kangwon, Korea	*E1*	*e1la*	37.8228	128.1555
Zhou et al. 2015	PI 578341	Khabarovsk, Russian Federatio	*E1*	*e1la*	48.4814433	135.072067
Zhou et al. 2015	PI 464927A	Liaoning, China	*E1*	*e1la*	41.9436543	122.529038
Zhou et al. 2015	PI 468916	Liaoning, China	*E1*	*e1la*	41.9436543	122.529038
Zhou et al. 2015	PI 522226	Primorye, Russian Federation	*E1*	*e1la*	45.0525641	135
Zhou et al. 2015	PI 366120	Akita, Japan	*E1*	*E1La*	39.7199668	140.10348
Zhou et al. 2015	PI 407027	Akita, Japan	*E1*	*E1La*	39.7199668	140.10348
Zhou et al. 2015	PI 504286	ChungchongPuk, Korea	*E1*	*E1La*	36.8	127.7
Zhou et al. 2015	PI 366121	Fukushima, Japan	*E1*	*E1La*	37.7607991	140.474786
Zhou et al. 2015	PI 458536	Heilongjiang, China	*E1*	*E1La*	47.1216472	128.738231
Zhou et al. 2015	PI 479769	Heilongjiang, China	*E1*	*E1La*	47.1216472	128.738231
Zhou et al. 2015	PI 522182B	Heilongjiang, China	*E1*	*E1La*	47.1216472	128.738231
Zhou et al. 2015	PI 593983	Hokkaido, Japan	*E1*	*E1La*	43.2203266	142.863474
Zhou et al. 2015	PI 366123	Iwate, Japan	*E1*	*E1La*	39.5832989	141.253457
Zhou et al. 2015	PI 378692	Iwate, Japan	*E1*	*E1La*	39.5832989	141.253457
Zhou et al. 2015	PI 562559	Jeonbuk, Korea	*E1*	*E1La*	35.7175	127.153
Zhou et al. 2015	PI 562565	Jeonbuk, Korea	*E1*	*E1La*	35.7175	127.153
Zhou et al. 2015	PI 464935	Jiangsu, China	*E1*	*E1La*	33.1401715	119.788925
Zhou et al. 2015	PI 507662	Kagoshima, Japan	*E1*	*E1La*	31.5968539	130.557139
Zhou et al. 2015	PI 407285	Kanagawa, Japan	*E1*	*E1La*	35.4913535	139.284143
Zhou et al. 2015	PI 407131	Kumamoto, Japan	*E1*	*E1La*	32.8032164	130.707937
Zhou et al. 2015	PI 407170	Kyonggi, Korea	*E1*	*E1La*	37.4138	127.5183
Zhou et al. 2015	PI 407246	KyongsangPuk, Korea	*E1*	*E1La*	36.0418465	129.365645
Zhou et al. 2015	PI 464929A	Liaoning, China	*E1*	*E1La*	41.9436543	122.529038
Zhou et al. 2015	PI 464929B	Liaoning, China	*E1*	*E1La*	41.9436543	122.529038
Zhou et al. 2015	PI 483460B	Liaoning, China	*E1*	*E1La*	41.9436543	122.529038
Zhou et al. 2015	PI 468400A	Ningxia, China	*E1*	*E1La*	37.198731	106.158094
Zhou et al. 2015	PI 483464A	Ningxia, China	*E1*	*E1La*	37.198731	106.158094
Zhou et al. 2015	PI 326582A	Primorye, Russian Federation	*E1*	*E1La*	45.0525641	135
Zhou et al. 2015	PI 522216	Primorye, Russian Federation	*E1*	*E1La*	45.0525641	135
Zhou et al. 2015	PI 522228	Primorye, Russian Federation	*E1*	*E1La*	45.0525641	135
Zhou et al. 2015	PI 483465	Shaanxi, China	*E1*	*E1La*	35.3939908	109.188005
Zhou et al. 2015	PI 549046	Shaanxi, China	*E1*	*E1La*	35.3939908	109.188005
Zhou et al. 2015	PI 597459C	Shandong, China	*E1*	*E1La*	35.8939566	117.9249
Zhou et al. 2015	PI 597459D	Shandong, China	*E1*	*E1La*	35.8939566	117.9249
Zhou et al. 2015	PI 597461A	Shandong, China	*E1*	*E1La*	35.8939566	117.9249
Zhou et al. 2015	PI 597461C	Shandong, China	*E1*	*E1La*	35.8939566	117.9249
Zhou et al. 2015	PI 407301	Zhejiang, China	*E1*	*E1La*	29.1416432	119.788925
Zhou et al. 2015	ZJ-Y108	Zhejiang, China	*E1*	*E1La*	29.1416432	119.788925
Zhou et al. 2015	ZJ-Y155	Zhejiang, China	*E1*	*E1La*	29.1416432	119.788925
Zhou et al. 2015	ZJ-Y188	Zhejiang, China	*E1*	*E1La*	29.1416432	119.788925
Zhou et al. 2015	ZJ-Y191	Zhejiang, China	*E1*	*E1La*	29.1416432	119.788925
Zhou et al. 2015	ZJ-Y200	Zhejiang, China	*E1*	*E1La*	29.1416432	119.788925
Zhou et al. 2015	ZJ-Y217	Zhejiang, China	*E1*	*E1La*	29.1416432	119.788925
Zhou et al. 2015	ZJ-Y2300-1	Zhejiang, China	*E1*	*E1La*	29.1416432	119.788925
Zhou et al. 2015	ZJ-Y282	Zhejiang, China	*E1*	*E1La*	29.1416432	119.788925
Zhou et al. 2015	ZJ-Y314	Zhejiang, China	*E1*	*E1La*	29.1416432	119.788925
Zhou et al. 2015	ZJ-YJ038	Zhejiang, China	*E1*	*E1La*	29.1416432	119.788925
Zhou et al. 2015	ZJ-YJ086	Zhejiang, China	*E1*	*E1La*	29.1416432	119.788925
Zhou et al. 2015	ZJ-ZY020	Zhejiang, China	*E1*	*E1La*	29.1416432	119.788925
Zhou et al. 2015	PI 578357	Amur, Russian Federation	*e1-as*	*E1La*	54.6035065	127.480172

*Latitude and longitude coordinates for each accession were obtained from the GRIN, where available. Where unavailable, coordinates for state/province of origin were obtained from Google geocoding.

Supplemental Table 6. Origin information for geographic assessment of North American cultivars

Accession	Cultivar Name	Origin	*E1* †	*E1La* †	Latitude*	Longitude*
PI 567179	'Envy'	Maine, United States	*E1*	*e1la:K82E*	45.253783	-69.4454689
FC 30689	Selection No. 9	Manitoba, Canada	*E1*	*e1la:K82E*	53.7609	-98.8139
PI 548395	'Ogemaw'	Michigan, United States	*E1*	*e1la:K82E*	44.3148443	-85.6023643
PI 607835	'UM3'	Minnesota, United States	*E1*	*e1la:K82E*	46.729553	-94.6858998
PI 548648	'Canatto'	Ontario, Canada	*E1*	*e1la:K82E*	51.253775	-85.323214
PI 548650	'Nattosan'	Ontario, Canada	*E1*	*e1la:K82E*	51.253775	-85.323214
PI 531068	'Stonewall'	Alabama, United States	*E1*	*E1La*	32.3182	-86.9023
PI 584506	'Carver'	Alabama, United States	*E1*	*E1La*	32.3182	-86.9023
PI 608033	'Kuell'	Alabama, United States	*E1*	*E1La*	32.3182	-86.9023
PI 548440	'Armredo'	Arizona, United States	*E1*	*E1La*	34.0489281	-111.093731
PI 567791	'Kino'	Arizona, United States	*E1*	*E1La*	34.0489281	-111.093731
PI 527704	'A6785'	Arkansas, United States	*E1*	*E1La*	35.20105	-91.8318334
PI 533602	'Lloyd'	Arkansas, United States	*E1*	*E1La*	35.20105	-91.8318334
PI 544354	'Walters'	Arkansas, United States	*E1*	*E1La*	35.20105	-91.8318334
PI 548658	'Lee 74'	Arkansas, United States	*E1*	*E1La*	35.20105	-91.8318334
PI 548695	'Dortchsoy 31'	Arkansas, United States	*E1*	*E1La*	35.20105	-91.8318334
PI 548696	'Dortchsoy 67'	Arkansas, United States	*E1*	*E1La*	35.20105	-91.8318334
PI 553039	'Davis'	Arkansas, United States	*E1*	*E1La*	35.20105	-91.8318334
PI 553040	'Jeff'	Arkansas, United States	*E1*	*E1La*	35.20105	-91.8318334
PI 553052	'Narow'	Arkansas, United States	*E1*	*E1La*	35.20105	-91.8318334
PI 559369	'Lee 68'	Arkansas, United States	*E1*	*E1La*	35.20105	-91.8318334
PI 559370	'Mack'	Arkansas, United States	*E1*	*E1La*	35.20105	-91.8318334
PI 559371	'Hood 75'	Arkansas, United States	*E1*	*E1La*	35.20105	-91.8318334
PI 593653	'Crowley'	Arkansas, United States	*E1*	*E1La*	35.20105	-91.8318334
PI 615582	'CAVINESS'	Arkansas, United States	*E1*	*E1La*	35.20105	-91.8318334
PI 619232	'UARK-5896'	Arkansas, United States	*E1*	*E1La*	35.20105	-91.8318334
PI 633609	'Lonoke'	Arkansas, United States	*E1*	*E1La*	35.20105	-91.8318334
PI 633610	'Desha'	Arkansas, United States	*E1*	*E1La*	35.20105	-91.8318334
PI 633970	'OZARK'	Arkansas, United States	*E1*	*E1La*	35.20105	-91.8318334
PI 639187	'UA 4805'	Arkansas, United States	*E1*	*E1La*	35.20105	-91.8318334
PI 648270	'OSAGE'	Arkansas, United States	*E1*	*E1La*	35.20105	-91.8318334
PI 87457	NA	British Columbia, Canada	*E1*	*E1La*	53.7266683	-127.647621
PI 548514	'Bethel'	Delaware, United States	*E1*	*E1La*	38.9108	-75.5277
PI 548548	'Delmar'	Delaware, United States	*E1*	*E1La*	38.9108	-75.5277
PI 548559	'Emerald'	Delaware, United States	*E1*	*E1La*	38.9108	-75.5277
PI 548624	'Verde'	Delaware, United States	*E1*	*E1La*	38.9108	-75.5277
PI 612608	'Celest'	Delaware, United States	*E1*	*E1La*	38.9108	-75.5277
PI 87165	NA	District of Columbia, United States	*E1*	*E1La*	38.9071923	-77.0368707
PI 87167	NA	District of Columbia, United States	*E1*	*E1La*	38.9071923	-77.0368707
PI 548659	'Braxton'	Florida, United States	*E1*	*E1La*	27.6648274	-81.5157535
PI 548660	'Bragg'	Florida, United States	*E1*	*E1La*	27.6648274	-81.5157535
PI 548662	'Hutton'	Florida, United States	*E1*	*E1La*	27.6648274	-81.5157535
PI 548664	'Cobb'	Florida, United States	*E1*	*E1La*	27.6648274	-81.5157535
PI 548665	'Kirby'	Florida, United States	*E1*	*E1La*	27.6648274	-81.5157535
PI 548666	'Hardee'	Florida, United States	*E1*	*E1La*	27.6648274	-81.5157535
PI 548969	'Alamo'	Florida, United States	*E1*	*E1La*	27.6648274	-81.5157535
PI 548970	'Foster'	Florida, United States	*E1*	*E1La*	27.6648274	-81.5157535
PI 548971	'Howard'	Florida, United States	*E1*	*E1La*	27.6648274	-81.5157535
PI 548972	'Jupiter'	Florida, United States	*E1*	*E1La*	27.6648274	-81.5157535
PI 548973	'Jupiter-R'	Florida, United States	*E1*	*E1La*	27.6648274	-81.5157535
PI 629015	'Hinson Long Juvenile'	Florida, United States	*E1*	*E1La*	27.6648274	-81.5157535
PI 511813	'Twiggs'	Georgia, United States	*E1*	*E1La*	32.1656221	-82.9000751
PI 522236	'Thomas'	Georgia, United States	*E1*	*E1La*	32.1656221	-82.9000751
PI 536009	'Colquitt'	Georgia, United States	*E1*	*E1La*	32.1656221	-82.9000751
PI 542712	'Bryan'	Georgia, United States	*E1*	*E1La*	32.1656221	-82.9000751
PI 553041	'Duocrop'	Georgia, United States	*E1*	*E1La*	32.1656221	-82.9000751
PI 553042	'Wright'	Georgia, United States	*E1*	*E1La*	32.1656221	-82.9000751
PI 553045	'Cook'	Georgia, United States	*E1*	*E1La*	32.1656221	-82.9000751
PI 553046	'Gasoy 17'	Georgia, United States	*E1*	*E1La*	32.1656221	-82.9000751
PI 553047	'Gordon'	Georgia, United States	*E1*	*E1La*	32.1656221	-82.9000751
PI 572238	'Haskell'	Georgia, United States	*E1*	*E1La*	32.1656221	-82.9000751
PI 576154	'Doles'	Georgia, United States	*E1*	*E1La*	32.1656221	-82.9000751
PI 595645	'Benning'	Georgia, United States	*E1*	*E1La*	32.1656221	-82.9000751
PI 602597	'Boggs'	Georgia, United States	*E1*	*E1La*	32.1656221	-82.9000751
PI 612157	'PRICHARD'	Georgia, United States	*E1*	*E1La*	32.1656221	-82.9000751
PI 355067	'Kahala'	Hawaii, United States	*E1*	*E1La*	19.8968	-155.5828
PI 355068	'Kaikoo'	Hawaii, United States	*E1*	*E1La*	19.8968	-155.5828
PI 355069	'Kailua'	Hawaii, United States	*E1*	*E1La*	19.8968	-155.5828
PI 355070	'Mokapu Summer'	Hawaii, United States	*E1*	*E1La*	19.8968	-155.5828
FC 31579	Illinois No. 893-645-431	Illinois, United States	*E1*	*E1La*	40.6331249	-89.3985283
PI 506417	'Egyptian'	Illinois, United States	*E1*	*E1La*	40.6331249	-89.3985283
PI 518676	'CN210'	Illinois, United States	*E1*	*E1La*	40.6331249	-89.3985283
PI 542045	'IL1'	Illinois, United States	*E1*	*E1La*	40.6331249	-89.3985283
PI 542046	'IL2'	Illinois, United States	*E1*	*E1La*	40.6331249	-89.3985283
PI 548314	'Chief'	Illinois, United States	*E1*	*E1La*	40.6331249	-89.3985283
PI 548645	'Pharaoh'	Illinois, United States	*E1*	*E1La*	40.6331249	-89.3985283
PI 548669	'Carlin'	Illinois, United States	*E1*	*E1La*	40.6331249	-89.3985283
PI 548672	'Little Wonder'	Illinois, United States	*E1*	*E1La*	40.6331249	-89.3985283
PI 548675	'Roe'	Illinois, United States	*E1*	*E1La*	40.6331249	-89.3985283
PI 553051	'Spry'	Illinois, United States	*E1*	*E1La*	40.6331249	-89.3985283
PI 604100	'LS90-1920'	Illinois, United States	*E1*	*E1La*	40.6331249	-89.3985283
PI 634335	'LS94-3207'	Illinois, United States	*E1*	*E1La*	40.6331249	-89.3985283

PI 548330	'Gibson'	Indiana, United States	*E1*	*E1La*	40.2671941	-86.1349019
PI 548503	'Adelphia'	Indiana, United States	*E1*	*E1La*	40.2671941	-86.1349019
PI 548510	'Beeson'	Indiana, United States	*E1*	*E1La*	40.2671941	-86.1349019
PI 548511	'Beeson 80'	Indiana, United States	*E1*	*E1La*	40.2671941	-86.1349019
PI 548513	'Wells II'	Indiana, United States	*E1*	*E1La*	40.2671941	-86.1349019
PI 548528	'Protana'	Indiana, United States	*E1*	*E1La*	40.2671941	-86.1349019
PI 548583	'Keller'	Indiana, United States	*E1*	*E1La*	40.2671941	-86.1349019
PI 548584	'Miami'	Indiana, United States	*E1*	*E1La*	40.2671941	-86.1349019
PI 548626	'Wabash'	Indiana, United States	*E1*	*E1La*	40.2671941	-86.1349019
PI 548630	'Wells'	Indiana, United States	*E1*	*E1La*	40.2671941	-86.1349019
PI 548691	'Agripro 26'	Indiana, United States	*E1*	*E1La*	40.2671941	-86.1349019
PI 548693	'Marshall'	Indiana, United States	*E1*	*E1La*	40.2671941	-86.1349019
PI 539866	'LS201'	Iowa, United States	*E1*	*E1La*	41.878	-93.0977
PI 543855	'Newton'	Iowa, United States	*E1*	*E1La*	41.878	-93.0977
PI 548303	'Bansei [Ames]'	Iowa, United States	*E1*	*E1La*	41.878	-93.0977
PI 548502	'Adams'	Iowa, United States	*E1*	*E1La*	41.878	-93.0977
PI 548516	'Blackhawk'	Iowa, United States	*E1*	*E1La*	41.878	-93.0977
PI 548521	'BSR 201'	Iowa, United States	*E1*	*E1La*	41.878	-93.0977
PI 548537	'Marion'	Iowa, United States	*E1*	*E1La*	41.878	-93.0977
PI 548550	'Disoy'	Iowa, United States	*E1*	*E1La*	41.878	-93.0977
PI 548552	'Kanrich'	Iowa, United States	*E1*	*E1La*	41.878	-93.0977
PI 548553	'Magna'	Iowa, United States	*E1*	*E1La*	41.878	-93.0977
PI 548554	'Prize'	Iowa, United States	*E1*	*E1La*	41.878	-93.0977
PI 548587	'Kim'	Iowa, United States	*E1*	*E1La*	41.878	-93.0977
PI 548676	'Bombay'	Iowa, United States	*E1*	*E1La*	41.878	-93.0977
PI 559934	'KS5292'	Kansas, United States	*E1*	*E1La*	39.0119	-98.4842
PI 595081	'KS4895'	Kansas, United States	*E1*	*E1La*	39.0119	-98.4842
PI 602950	'KS4997'	Kansas, United States	*E1*	*E1La*	39.0119	-98.4842
PI 576440	'Calhoun'	Kentucky, United States	*E1*	*E1La*	37.8393332	-84.2700179
PI 590931	'CF492'	Kentucky, United States	*E1*	*E1La*	37.8393332	-84.2700179
PI 596540	'Camp-lx2'	Kentucky, United States	*E1*	*E1La*	37.8393332	-84.2700179
PI 510675	'Gregg'	Louisiana, United States	*E1*	*E1La*	30.9842977	-91.9623327
PI 543832	'Buckshot 723'	Louisiana, United States	*E1*	*E1La*	30.9842977	-91.9623327
PI 548436	'Acadian'	Louisiana, United States	*E1*	*E1La*	30.9842977	-91.9623327
PI 548461	'Improved Pelican'	Louisiana, United States	*E1*	*E1La*	30.9842977	-91.9623327
PI 548465	'Louisiana Green'	Louisiana, United States	*E1*	*E1La*	30.9842977	-91.9623327
PI 548476	'Nela'	Louisiana, United States	*E1*	*E1La*	30.9842977	-91.9623327
PI 567788	'Bienville'	Louisiana, United States	*E1*	*E1La*	30.9842977	-91.9623327
PI 567789	'Bossier'	Louisiana, United States	*E1*	*E1La*	30.9842977	-91.9623327
PI 567790	'Curtis'	Louisiana, United States	*E1*	*E1La*	30.9842977	-91.9623327
FC 30691	NA	Manitoba, Canada	*E1*	*E1La*	53.7609	-98.8139
PI 548504	'Altona'	Manitoba, Canada	*E1*	*E1La*	53.7609	-98.8139
PI 559932	'Manokin'	Maryland, United States	*E1*	*E1La*	39.0457549	-76.6412712
PI 601982	'Derry'	Maryland, United States	*E1*	*E1La*	39.0457549	-76.6412712
PI 601984	'Tyrone'	Maryland, United States	*E1*	*E1La*	39.0457549	-76.6412712
PI 632905	'Moon Cake'	Maryland, United States	*E1*	*E1La*	39.0457549	-76.6412712
PI 655521	'GREENCASTLE'	Maryland, United States	*E1*	*E1La*	39.0457549	-76.6412712
PI 572245	'Felix'	Michigan, United States	*E1*	*E1La*	44.3148443	-85.6023643
PI 602059	'Apollo'	Michigan, United States	*E1*	*E1La*	44.3148443	-85.6023643
PI 508083	'Dassel'	Minnesota, United States	*E1*	*E1La*	46.729553	-94.6858998
PI 508084	'Sibley'	Minnesota, United States	*E1*	*E1La*	46.729553	-94.6858998
PI 537096	'Minnatto'	Minnesota, United States	*E1*	*E1La*	46.729553	-94.6858998
PI 542402	'Chico'	Minnesota, United States	*E1*	*E1La*	46.729553	-94.6858998
PI 542403	'Dawson'	Minnesota, United States	*E1*	*E1La*	46.729553	-94.6858998
PI 542404	'Ozzie'	Minnesota, United States	*E1*	*E1La*	46.729553	-94.6858998
PI 548501	'Wilkin'	Minnesota, United States	*E1*	*E1La*	46.729553	-94.6858998
PI 548560	'Evans'	Minnesota, United States	*E1*	*E1La*	46.729553	-94.6858998
PI 548615	'Simpson'	Minnesota, United States	*E1*	*E1La*	46.729553	-94.6858998
PI 548620	'Steele'	Minnesota, United States	*E1*	*E1La*	46.729553	-94.6858998
PI 562372	'Agassiz'	Minnesota, United States	*E1*	*E1La*	46.729553	-94.6858998
PI 592523	'Glacier'	Minnesota, United States	*E1*	*E1La*	46.729553	-94.6858998
PI 592524	'Granite'	Minnesota, United States	*E1*	*E1La*	46.729553	-94.6858998
PI 592560	'Toyopro'	Minnesota, United States	*E1*	*E1La*	46.729553	-94.6858998
PI 602594	'MN0301'	Minnesota, United States	*E1*	*E1La*	46.729553	-94.6858998
PI 612764	'MN0901'	Minnesota, United States	*E1*	*E1La*	46.729553	-94.6858998
PI 629005	'MN0302'	Minnesota, United States	*E1*	*E1La*	46.729553	-94.6858998
PI 515960	'Sharkey'	Mississippi, United States	*E1*	*E1La*	32.3546679	-89.3985283
PI 533604	'Lamar'	Mississippi, United States	*E1*	*E1La*	32.3546679	-89.3985283
PI 533605	'Cordell'	Mississippi, United States	*E1*	*E1La*	32.3546679	-89.3985283
PI 548468	'Mamloxi'	Mississippi, United States	*E1*	*E1La*	32.3546679	-89.3985283
PI 548470	'Mamotan 6640'	Mississippi, United States	*E1*	*E1La*	32.3546679	-89.3985283
PI 548471	'Mamredo'	Mississippi, United States	*E1*	*E1La*	32.3546679	-89.3985283
PI 548653	'Dorman'	Mississippi, United States	*E1*	*E1La*	32.3546679	-89.3985283
PI 548654	'Hill'	Mississippi, United States	*E1*	*E1La*	32.3546679	-89.3985283
PI 548655	'Forrest'	Mississippi, United States	*E1*	*E1La*	32.3546679	-89.3985283
PI 548656	'Lee'	Mississippi, United States	*E1*	*E1La*	32.3546679	-89.3985283
PI 548661	'Semmes'	Mississippi, United States	*E1*	*E1La*	32.3546679	-89.3985283
PI 548975	'Centennial'	Mississippi, United States	*E1*	*E1La*	32.3546679	-89.3985283
PI 548976	'Dyer'	Mississippi, United States	*E1*	*E1La*	32.3546679	-89.3985283
PI 548977	'Epps'	Mississippi, United States	*E1*	*E1La*	32.3546679	-89.3985283
PI 548979	'Govan'	Mississippi, United States	*E1*	*E1La*	32.3546679	-89.3985283
PI 548980	'Hood'	Mississippi, United States	*E1*	*E1La*	32.3546679	-89.3985283
PI 548981	'Leflore'	Mississippi, United States	*E1*	*E1La*	32.3546679	-89.3985283
PI 548982	'Pickett 71'	Mississippi, United States	*E1*	*E1La*	32.3546679	-89.3985283
PI 548983	'Tracy'	Mississippi, United States	*E1*	*E1La*	32.3546679	-89.3985283
PI 548984	'Tracy-M'	Mississippi, United States	*E1*	*E1La*	32.3546679	-89.3985283
PI 564261	'Vernal'	Mississippi, United States	*E1*	*E1La*	32.3546679	-89.3985283

PI 576857	'LYON'	Mississippi, United States	*E1*	*E1La*	32.3546679	-89.3985283
PI 602496	'Pace'	Mississippi, United States	*E1*	*E1La*	32.3546679	-89.3985283
PI 612146	'BOLIVAR'	Mississippi, United States	*E1*	*E1La*	32.3546679	-89.3985283
PI 613195	'Fowler'	Mississippi, United States	*E1*	*E1La*	32.3546679	-89.3985283
PI 636463	'Freedom'	Mississippi, United States	*E1*	*E1La*	32.3546679	-89.3985283
PI 543794	'Delsoy 4900'	Missouri, United States	*E1*	*E1La*	37.9642529	-91.8318334
PI 543795	'Hartwig'	Missouri, United States	*E1*	*E1La*	37.9642529	-91.8318334
PI 548604	'Pershing'	Missouri, United States	*E1*	*E1La*	37.9642529	-91.8318334
PI 556738	'Bradley'	Missouri, United States	*E1*	*E1La*	37.9642529	-91.8318334
PI 561400	'Rhodes'	Missouri, United States	*E1*	*E1La*	37.9642529	-91.8318334
PI 595765	'Delsoy 5500'	Missouri, United States	*E1*	*E1La*	37.9642529	-91.8318334
PI 607528	'Delsoy 5710'	Missouri, United States	*E1*	*E1La*	37.9642529	-91.8318334
PI 614732	'Anand'	Missouri, United States	*E1*	*E1La*	37.9642529	-91.8318334
PI 635039	'S99-3181'	Missouri, United States	*E1*	*E1La*	37.9642529	-91.8318334
PI 548564	'Fremont'	Nebraska, United States	*E1*	*E1La*	41.4925374	-99.9018131
PI 548566	'Nebsoy'	Nebraska, United States	*E1*	*E1La*	41.4925374	-99.9018131
PI 583835	'Mercury'	Nebraska, United States	*E1*	*E1La*	41.4925374	-99.9018131
PI 583837	'Saturn'	Nebraska, United States	*E1*	*E1La*	41.4925374	-99.9018131
PI 508266	'Young'	North Carolina, United States	*E1*	*E1La*	35.7595731	-79.0192997
PI 508267	'Johnston'	North Carolina, United States	*E1*	*E1La*	35.7595731	-79.0192997
PI 548657	'Jackson'	North Carolina, United States	*E1*	*E1La*	35.7595731	-79.0192997
PI 548986	'Brim'	North Carolina, United States	*E1*	*E1La*	35.7595731	-79.0192997
PI 548987	'Dare'	North Carolina, United States	*E1*	*E1La*	35.7595731	-79.0192997
PI 548988	'Pickett'	North Carolina, United States	*E1*	*E1La*	35.7595731	-79.0192997
PI 548989	'Ransom'	North Carolina, United States	*E1*	*E1La*	35.7595731	-79.0192997
PI 572239	'Holladay'	North Carolina, United States	*E1*	*E1La*	35.7595731	-79.0192997
PI 583367	'Pearl'	North Carolina, United States	*E1*	*E1La*	35.7595731	-79.0192997
PI 594922	'Graham'	North Carolina, United States	*E1*	*E1La*	35.7595731	-79.0192997
PI 596414	'Clifford'	North Carolina, United States	*E1*	*E1La*	35.7595731	-79.0192997
PI 597389	'Prolina'	North Carolina, United States	*E1*	*E1La*	35.7595731	-79.0192997
PI 614702	'Soyola'	North Carolina, United States	*E1*	*E1La*	35.7595731	-79.0192997
PI 615694	'N7001'	North Carolina, United States	*E1*	*E1La*	35.7595731	-79.0192997
PI 615695	'N7103'	North Carolina, United States	*E1*	*E1La*	35.7595731	-79.0192997
PI 617045	'NC-Roy'	North Carolina, United States	*E1*	*E1La*	35.7595731	-79.0192997
PI 619615	'N6201'	North Carolina, United States	*E1*	*E1La*	35.7595731	-79.0192997
PI 619616	'N7101'	North Carolina, United States	*E1*	*E1La*	35.7595731	-79.0192997
PI 619617	'N7102'	North Carolina, United States	*E1*	*E1La*	35.7595731	-79.0192997
PI 641156	'NC-Raleigh'	North Carolina, United States	*E1*	*E1La*	35.7595731	-79.0192997
PI 642732	'Nitrasoy'	North Carolina, United States	*E1*	*E1La*	35.7595731	-79.0192997
PI 647085	'N7002'	North Carolina, United States	*E1*	*E1La*	35.7595731	-79.0192997
PI 647086	'N8001'	North Carolina, United States	*E1*	*E1La*	35.7595731	-79.0192997
PI 654355	'N8101'	North Carolina, United States	*E1*	*E1La*	35.7595731	-79.0192997
PI 658498	'N6202'	North Carolina, United States	*E1*	*E1La*	35.7595731	-79.0192997
PI 587091	'Council'	North Dakota, United States	*E1*	*E1La*	47.5514926	-101.002012
PI 596541	'Traill'	North Dakota, United States	*E1*	*E1La*	47.5514926	-101.002012
PI 602896	'Daksoy'	North Dakota, United States	*E1*	*E1La*	47.5514926	-101.002012
PI 602897	'Jim'	North Dakota, United States	*E1*	*E1La*	47.5514926	-101.002012
PI 603900	'Norpro'	North Dakota, United States	*E1*	*E1La*	47.5514926	-101.002012
PI 614831	'Barnes'	North Dakota, United States	*E1*	*E1La*	47.5514926	-101.002012
PI 615585	'Sargent'	North Dakota, United States	*E1*	*E1La*	47.5514926	-101.002012
PI 615586	'Walsh'	North Dakota, United States	*E1*	*E1La*	47.5514926	-101.002012
PI 638510	'Pembina'	North Dakota, United States	*E1*	*E1La*	47.5514926	-101.002012
PI 638511	'ProSoy'	North Dakota, United States	*E1*	*E1La*	47.5514926	-101.002012
PI 536636	'Ripley'	Ohio, United States	*E1*	*E1La*	40.4173	-82.9071
PI 548387	'Miller 67'	Ohio, United States	*E1*	*E1La*	40.4173	-82.9071
PI 548990	'Sohoma'	Oklahoma, United States	*E1*	*E1La*	35.0077519	-97.092877
PI 553050	'Choska'	Oklahoma, United States	*E1*	*E1La*	35.0077519	-97.092877
PI 618808	'Catoosa'	Oklahoma, United States	*E1*	*E1La*	35.0077519	-97.092877
PI 618809	'Washita'	Oklahoma, United States	*E1*	*E1La*	35.0077519	-97.092877
PI 548515	'Bicentennial'	Ontario, Canada	*E1*	*E1La*	51.253775	-85.323214
PI 548545	'Merit'	Ontario, Canada	*E1*	*E1La*	51.253775	-85.323214
PI 548571	'Harlon'	Ontario, Canada	*E1*	*E1La*	51.253775	-85.323214
PI 548592	'Maple Amber'	Ontario, Canada	*E1*	*E1La*	51.253775	-85.323214
PI 548593	'Maple Arrow'	Ontario, Canada	*E1*	*E1La*	51.253775	-85.323214
PI 548594	'Maple Presto'	Ontario, Canada	*E1*	*E1La*	51.253775	-85.323214
PI 548595	'Maple Isle'	Ontario, Canada	*E1*	*E1La*	51.253775	-85.323214
PI 548596	'Maple Ridge'	Ontario, Canada	*E1*	*E1La*	51.253775	-85.323214
PI 548637	'OAC Aries'	Ontario, Canada	*E1*	*E1La*	51.253775	-85.323214
PI 548638	'OAC Libra'	Ontario, Canada	*E1*	*E1La*	51.253775	-85.323214
PI 548639	'OAC Pisces'	Ontario, Canada	*E1*	*E1La*	51.253775	-85.323214
PI 548640	'OAC Scorpio'	Ontario, Canada	*E1*	*E1La*	51.253775	-85.323214
PI 548643	'Maple Glen'	Ontario, Canada	*E1*	*E1La*	51.253775	-85.323214
PI 567782	'OAC Dorado'	Ontario, Canada	*E1*	*E1La*	51.253775	-85.323214
PI 567783	'OAC Eclipse'	Ontario, Canada	*E1*	*E1La*	51.253775	-85.323214
PI 567784	'OAC Frontier'	Ontario, Canada	*E1*	*E1La*	51.253775	-85.323214
PI 567787	'OAC Vision'	Ontario, Canada	*E1*	*E1La*	51.253775	-85.323214
PI 572242	'RCAT Angora'	Ontario, Canada	*E1*	*E1La*	51.253775	-85.323214
PI 548674	'Polysoy'	Pennsylvania, United States	*E1*	*E1La*	41.2033216	-77.1945247
PI 536637	'Perrin'	South Carolina, United States	*E1*	*E1La*	33.836081	-81.1637245
PI 548497	'Yelredo'	South Carolina, United States	*E1*	*E1La*	33.836081	-81.1637245
PI 548697	'Majos'	South Carolina, United States	*E1*	*E1La*	33.836081	-81.1637245
PI 548698	'Yelnanda'	South Carolina, United States	*E1*	*E1La*	33.836081	-81.1637245
PI 548985	'Kershaw'	South Carolina, United States	*E1*	*E1La*	33.836081	-81.1637245
PI 555453	'Hagood'	South Carolina, United States	*E1*	*E1La*	33.836081	-81.1637245
PI 568236	'Maxcy'	South Carolina, United States	*E1*	*E1La*	33.836081	-81.1637245
PI 592756	'Dillon'	South Carolina, United States	*E1*	*E1La*	33.836081	-81.1637245
PI 599333	'Musen'	South Carolina, United States	*E1*	*E1La*	33.836081	-81.1637245

PI 603953	'Motte'	South Carolina, United States	*E1*	*E1La*	33.836081	-81.1637245
PI 614156	'Hampton'	South Carolina, United States	*E1*	*E1La*	33.836081	-81.1637245
PI 617041	'Santee'	South Carolina, United States	*E1*	*E1La*	33.836081	-81.1637245
PI 633622	'CNS 4'	South Carolina, United States	*E1*	*E1La*	33.836081	-81.1637245
PI 548477	'Ogden'	Tennessee, United States	*E1*	*E1La*	35.5174913	-86.5804473
PI 548492	'Tennessee Non Pop'	Tennessee, United States	*E1*	*E1La*	35.5174913	-86.5804473
PI 548494	'Volstate'	Tennessee, United States	*E1*	*E1La*	35.5174913	-86.5804473
PI 548974	'Bedford'	Tennessee, United States	*E1*	*E1La*	35.5174913	-86.5804473
PI 548991	'TN 5-85'	Tennessee, United States	*E1*	*E1La*	35.5174913	-86.5804473
PI 564849	'NATHAN'	Tennessee, United States	*E1*	*E1La*	35.5174913	-86.5804473
PI 564999	'TN 6-90'	Tennessee, United States	*E1*	*E1La*	35.5174913	-86.5804473
PI 598358	'TN 5-95'	Tennessee, United States	*E1*	*E1La*	35.5174913	-86.5804473
PI 630984	'5601T'	Tennessee, United States	*E1*	*E1La*	35.5174913	-86.5804473
PI 634193	'5002T'	Tennessee, United States	*E1*	*E1La*	35.5174913	-86.5804473
PI 518665	'Padre'	Texas, United States	*E1*	*E1La*	31.9685988	-99.9018131
PI 535807	'Crockett'	Texas, United States	*E1*	*E1La*	31.9685988	-99.9018131
PI 548663	'Dowling'	Texas, United States	*E1*	*E1La*	31.9685988	-99.9018131
PI 548978	'Gail'	Texas, United States	*E1*	*E1La*	31.9685988	-99.9018131
PI 508268	'Toano'	Virginia, United States	*E1*	*E1La*	37.4315734	-78.6568942
PI 508269	'Stafford'	Virginia, United States	*E1*	*E1La*	37.4315734	-78.6568942
PI 518664	'Hutcheson'	Virginia, United States	*E1*	*E1La*	37.4315734	-78.6568942
PI 548458	'Harrel'	Virginia, United States	*E1*	*E1La*	37.4315734	-78.6568942
PI 548475	'Nansemond'	Virginia, United States	*E1*	*E1La*	37.4315734	-78.6568942
PI 548481	'Pine Dell Perfection'	Virginia, United States	*E1*	*E1La*	37.4315734	-78.6568942
PI 548483	'Pocahontas'	Virginia, United States	*E1*	*E1La*	37.4315734	-78.6568942
PI 548627	'Ware'	Virginia, United States	*E1*	*E1La*	37.4315734	-78.6568942
PI 548667	'Essex'	Virginia, United States	*E1*	*E1La*	37.4315734	-78.6568942
PI 553038	'York'	Virginia, United States	*E1*	*E1La*	37.4315734	-78.6568942
PI 553043	'Bay'	Virginia, United States	*E1*	*E1La*	37.4315734	-78.6568942
PI 553044	'Camp'	Virginia, United States	*E1*	*E1La*	37.4315734	-78.6568942
PI 553048	'Vance'	Virginia, United States	*E1*	*E1La*	37.4315734	-78.6568942
PI 553049	'Shore'	Virginia, United States	*E1*	*E1La*	37.4315734	-78.6568942
PI 583366	'Chesapeake'	Virginia, United States	*E1*	*E1La*	37.4315734	-78.6568942
PI 597388	'Accomac'	Virginia, United States	*E1*	*E1La*	37.4315734	-78.6568942
PI 633049	'ASMARA'	Virginia, United States	*E1*	*E1La*	37.4315734	-78.6568942
PI 633424	'Randolph'	Virginia, United States	*E1*	*E1La*	37.4315734	-78.6568942
PI 633567	'Owens'	Virginia, United States	*E1*	*E1La*	37.4315734	-78.6568942
PI 633621	Early Woods Yellow	Virginia, United States	*E1*	*E1La*	37.4315734	-78.6568942
PI 548600	'Morsoy'	Manitoba, Canada	*e1-as*	*e1la:K82E*	53.7609	-98.8139
PI 593655	'Danatto'	North Dakota, United States	*e1-as*	*e1la:K82E*	47.5514926	-101.002012
PI 631437	'Nornatto'	North Dakota, United States	*e1-as*	*e1la:K82E*	47.5514926	-101.002012
PI 631438	'Nannonatto'	North Dakota, United States	*e1-as*	*e1la:K82E*	47.5514926	-101.002012
PI 548398	'Pagoda'	Ontario, Canada	*e1-as*	*e1la:K82E*	51.253775	-85.323214
PI 548498	'Acme'	Ontario, Canada	*e1-as*	*e1la:K82E*	51.253775	-85.323214
PI 248398	Illinois 301	Illinois, United States	*e1-as*	*E1La*	40.6331249	-89.3985283
PI 248402	Manhatan	Illinois, United States	*e1-as*	*E1La*	40.6331249	-89.3985283
PI 512039	'Pyramid'	Illinois, United States	*e1-as*	*E1La*	40.6331249	-89.3985283
PI 518669	'Corsoy 79'	Illinois, United States	*e1-as*	*E1La*	40.6331249	-89.3985283
PI 518670	'Williams 79'	Illinois, United States	*e1-as*	*E1La*	40.6331249	-89.3985283
PI 518671	'Williams 82'	Illinois, United States	*e1-as*	*E1La*	40.6331249	-89.3985283
PI 518672	'Will'	Illinois, United States	*e1-as*	*E1La*	40.6331249	-89.3985283
PI 518673	'Lawrence'	Illinois, United States	*e1-as*	*E1La*	40.6331249	-89.3985283
PI 518674	'Fayette'	Illinois, United States	*e1-as*	*E1La*	40.6331249	-89.3985283
PI 518675	'Cartter'	Illinois, United States	*e1-as*	*E1La*	40.6331249	-89.3985283
PI 518677	'CN290'	Illinois, United States	*e1-as*	*E1La*	40.6331249	-89.3985283
PI 533654	'LN83-2356'	Illinois, United States	*e1-as*	*E1La*	40.6331249	-89.3985283
PI 533655	'Burlison'	Illinois, United States	*e1-as*	*E1La*	40.6331249	-89.3985283
PI 540554	'Bell'	Illinois, United States	*e1-as*	*E1La*	40.6331249	-89.3985283
PI 540555	'Hamilton'	Illinois, United States	*e1-as*	*E1La*	40.6331249	-89.3985283
PI 540556	'Jack'	Illinois, United States	*e1-as*	*E1La*	40.6331249	-89.3985283
PI 542043	'Linford'	Illinois, United States	*e1-as*	*E1La*	40.6331249	-89.3985283
PI 542044	'Kunitz'	Illinois, United States	*e1-as*	*E1La*	40.6331249	-89.3985283
PI 548362	'Lincoln'	Illinois, United States	*e1-as*	*E1La*	40.6331249	-89.3985283
PI 548421	'Viking'	Illinois, United States	*e1-as*	*E1La*	40.6331249	-89.3985283
PI 548530	'Chippewa'	Illinois, United States	*e1-as*	*E1La*	40.6331249	-89.3985283
PI 548531	'Chippewa 64'	Illinois, United States	*e1-as*	*E1La*	40.6331249	-89.3985283
PI 548532	'Clark 63'	Illinois, United States	*e1-as*	*E1La*	40.6331249	-89.3985283
PI 548533	'Clark'	Illinois, United States	*e1-as*	*E1La*	40.6331249	-89.3985283
PI 548556	'Elf'	Illinois, United States	*e1-as*	*E1La*	40.6331249	-89.3985283
PI 548563	'Franklin'	Illinois, United States	*e1-as*	*E1La*	40.6331249	-89.3985283
PI 548569	'Hack'	Illinois, United States	*e1-as*	*E1La*	40.6331249	-89.3985283
PI 548574	'Shelby'	Illinois, United States	*e1-as*	*E1La*	40.6331249	-89.3985283
PI 548575	'Harosoy 63'	Illinois, United States	*e1-as*	*E1La*	40.6331249	-89.3985283
PI 548578	'Hawkeye 63'	Illinois, United States	*e1-as*	*E1La*	40.6331249	-89.3985283
PI 548622	'Union'	Illinois, United States	*e1-as*	*E1La*	40.6331249	-89.3985283
PI 548628	'Wayne'	Illinois, United States	*e1-as*	*E1La*	40.6331249	-89.3985283
PI 548631	'Williams'	Illinois, United States	*e1-as*	*E1La*	40.6331249	-89.3985283
PI 548632	'Woodworth'	Illinois, United States	*e1-as*	*E1La*	40.6331249	-89.3985283
PI 548635	'Chamberlain'	Illinois, United States	*e1-as*	*E1La*	40.6331249	-89.3985283
PI 548671	'Fabulin'	Illinois, United States	*e1-as*	*E1La*	40.6331249	-89.3985283
PI 548677	'Ennis I'	Illinois, United States	*e1-as*	*E1La*	40.6331249	-89.3985283
PI 548681	'SRF 100'	Illinois, United States	*e1-as*	*E1La*	40.6331249	-89.3985283
PI 548682	'SRF 400'	Illinois, United States	*e1-as*	*E1La*	40.6331249	-89.3985283
PI 548683	'SRF 150'	Illinois, United States	*e1-as*	*E1La*	40.6331249	-89.3985283
PI 548684	'SRF 307B'	Illinois, United States	*e1-as*	*E1La*	40.6331249	-89.3985283
PI 548685	'SRF 450'	Illinois, United States	*e1-as*	*E1La*	40.6331249	-89.3985283
PI 548686	'SRF 300'	Illinois, United States	*e1-as*	*E1La*	40.6331249	-89.3985283

PI 572240	'Nile'	Illinois, United States	*e1-as*	*E1La*	40.6331249	-89.3985283
PI 574534	'Piatt'	Illinois, United States	*e1-as*	*E1La*	40.6331249	-89.3985283
PI 584441	'Yale'	Illinois, United States	*e1-as*	*E1La*	40.6331249	-89.3985283
PI 593256	'Cisne'	Illinois, United States	*e1-as*	*E1La*	40.6331249	-89.3985283
PI 593257	'LN90-4524'	Illinois, United States	*e1-as*	*E1La*	40.6331249	-89.3985283
PI 593258	'Macon'	Illinois, United States	*e1-as*	*E1La*	40.6331249	-89.3985283
PI 593259	'Iroquois'	Illinois, United States	*e1-as*	*E1La*	40.6331249	-89.3985283
PI 597381	'Savoy'	Illinois, United States	*e1-as*	*E1La*	40.6331249	-89.3985283
PI 597382	'Omaha'	Illinois, United States	*e1-as*	*E1La*	40.6331249	-89.3985283
PI 597383	'LN89-3264'	Illinois, United States	*e1-as*	*E1La*	40.6331249	-89.3985283
PI 597384	'LN89-3615'	Illinois, United States	*e1-as*	*E1La*	40.6331249	-89.3985283
PI 597385	'LN92-11008'	Illinois, United States	*e1-as*	*E1La*	40.6331249	-89.3985283
PI 597386	'Dwight'	Illinois, United States	*e1-as*	*E1La*	40.6331249	-89.3985283
PI 597387	'Pana'	Illinois, United States	*e1-as*	*E1La*	40.6331249	-89.3985283
PI 606748	'REND'	Illinois, United States	*e1-as*	*E1La*	40.6331249	-89.3985283
PI 606749	'INA'	Illinois, United States	*e1-as*	*E1La*	40.6331249	-89.3985283
PI 607380	'LS92-1800'	Illinois, United States	*e1-as*	*E1La*	40.6331249	-89.3985283
PI 607385	'LN92-7369'	Illinois, United States	*e1-as*	*E1La*	40.6331249	-89.3985283
PI 614088	'Loda'	Illinois, United States	*e1-as*	*E1La*	40.6331249	-89.3985283
PI 620883	'LS93-0375'	Illinois, United States	*e1-as*	*E1La*	40.6331249	-89.3985283
PI 633983	'LN97-15076'	Illinois, United States	*e1-as*	*E1La*	40.6331249	-89.3985283
PI 639740	'LD00-3309'	Illinois, United States	*e1-as*	*E1La*	40.6331249	-89.3985283
PI 525454	'Spencer'	Indiana, United States	*e1-as*	*E1La*	40.2671941	-86.1349019
PI 548507	'Amsoy 71'	Indiana, United States	*e1-as*	*E1La*	40.2671941	-86.1349019
PI 548512	'Century'	Indiana, United States	*e1-as*	*E1La*	40.2671941	-86.1349019
PI 548517	'Bonus'	Indiana, United States	*e1-as*	*E1La*	40.2671941	-86.1349019
PI 548518	'Cutler 71'	Indiana, United States	*e1-as*	*E1La*	40.2671941	-86.1349019
PI 548527	'Calland'	Indiana, United States	*e1-as*	*E1La*	40.2671941	-86.1349019
PI 548547	'Cutler'	Indiana, United States	*e1-as*	*E1La*	40.2671941	-86.1349019
PI 548585	'Winchester'	Indiana, United States	*e1-as*	*E1La*	40.2671941	-86.1349019
PI 548586	'Kent'	Indiana, United States	*e1-as*	*E1La*	40.2671941	-86.1349019
PI 548589	'Lindarin'	Indiana, United States	*e1-as*	*E1La*	40.2671941	-86.1349019
PI 548590	'Lindarin 63'	Indiana, United States	*e1-as*	*E1La*	40.2671941	-86.1349019
PI 548603	'Perry'	Indiana, United States	*e1-as*	*E1La*	40.2671941	-86.1349019
PI 548636	'Regal'	Indiana, United States	*e1-as*	*E1La*	40.2671941	-86.1349019
PI 548692	'AP 200'	Indiana, United States	*e1-as*	*E1La*	40.2671941	-86.1349019
PI 577798	'Bronson'	Indiana, United States	*e1-as*	*E1La*	40.2671941	-86.1349019
PI 587185	'Probst'	Indiana, United States	*e1-as*	*E1La*	40.2671941	-86.1349019
PI 595926	'Athow'	Indiana, United States	*e1-as*	*E1La*	40.2671941	-86.1349019
PI 657626	'CL0J095-4'	Indiana, United States	*e1-as*	*E1La*	40.2671941	-86.1349019
PI 509044	'Pella 86'	Iowa, United States	*e1-as*	*E1La*	41.878	-93.0977
PI 518667	'Harper 87'	Iowa, United States	*e1-as*	*E1La*	41.878	-93.0977
PI 525453	'Conrad'	Iowa, United States	*e1-as*	*E1La*	41.878	-93.0977
PI 537094	'Kenwood'	Iowa, United States	*e1-as*	*E1La*	41.878	-93.0977
PI 537095	'Marcus'	Iowa, United States	*e1-as*	*E1La*	41.878	-93.0977
PI 539860	'SS201'	Iowa, United States	*e1-as*	*E1La*	41.878	-93.0977
PI 539861	'SS202'	Iowa, United States	*e1-as*	*E1La*	41.878	-93.0977
PI 539862	'HP201'	Iowa, United States	*e1-as*	*E1La*	41.878	-93.0977
PI 539863	'HP202'	Iowa, United States	*e1-as*	*E1La*	41.878	-93.0977
PI 539864	'HP203'	Iowa, United States	*e1-as*	*E1La*	41.878	-93.0977
PI 539865	'HP204'	Iowa, United States	*e1-as*	*E1La*	41.878	-93.0977
PI 539867	'LS301'	Iowa, United States	*e1-as*	*E1La*	41.878	-93.0977
PI 546487	'Archer'	Iowa, United States	*e1-as*	*E1La*	41.878	-93.0977
PI 548304	'Bavender Special A'	Iowa, United States	*e1-as*	*E1La*	41.878	-93.0977
PI 548305	'Bavender Special B'	Iowa, United States	*e1-as*	*E1La*	41.878	-93.0977
PI 548306	'Bavender Special C'	Iowa, United States	*e1-as*	*E1La*	41.878	-93.0977
PI 548506	'Amsoy'	Iowa, United States	*e1-as*	*E1La*	41.878	-93.0977
PI 548519	'BSR 101'	Iowa, United States	*e1-as*	*E1La*	41.878	-93.0977
PI 548520	'Preston'	Iowa, United States	*e1-as*	*E1La*	41.878	-93.0977
PI 548522	'BSR 301'	Iowa, United States	*e1-as*	*E1La*	41.878	-93.0977
PI 548523	'Pella'	Iowa, United States	*e1-as*	*E1La*	41.878	-93.0977
PI 548524	'Weber'	Iowa, United States	*e1-as*	*E1La*	41.878	-93.0977
PI 548525	'BSR 302'	Iowa, United States	*e1-as*	*E1La*	41.878	-93.0977
PI 548526	'Hardin'	Iowa, United States	*e1-as*	*E1La*	41.878	-93.0977
PI 548536	'Coles'	Iowa, United States	*e1-as*	*E1La*	41.878	-93.0977
PI 548540	'Corsoy'	Iowa, United States	*e1-as*	*E1La*	41.878	-93.0977
PI 548542	'Cumberland'	Iowa, United States	*e1-as*	*E1La*	41.878	-93.0977
PI 548543	'Oakland'	Iowa, United States	*e1-as*	*E1La*	41.878	-93.0977
PI 548551	'Hark'	Iowa, United States	*e1-as*	*E1La*	41.878	-93.0977
PI 548557	'Elgin'	Iowa, United States	*e1-as*	*E1La*	41.878	-93.0977
PI 548558	'Harper'	Iowa, United States	*e1-as*	*E1La*	41.878	-93.0977
PI 548562	'Ford'	Iowa, United States	*e1-as*	*E1La*	41.878	-93.0977
PI 548577	'Hawkeye'	Iowa, United States	*e1-as*	*E1La*	41.878	-93.0977
PI 548588	'Lakota'	Iowa, United States	*e1-as*	*E1La*	41.878	-93.0977
PI 548608	'Provar'	Iowa, United States	*e1-as*	*E1La*	41.878	-93.0977
PI 548609	'Rampage'	Iowa, United States	*e1-as*	*E1La*	41.878	-93.0977
PI 548610	'Wirth'	Iowa, United States	*e1-as*	*E1La*	41.878	-93.0977
PI 548616	'Sloan'	Iowa, United States	*e1-as*	*E1La*	41.878	-93.0977
PI 548617	'Vickery'	Iowa, United States	*e1-as*	*E1La*	41.878	-93.0977
PI 548618	'Vinton'	Iowa, United States	*e1-as*	*E1La*	41.878	-93.0977
PI 548625	'Vinton 81'	Iowa, United States	*e1-as*	*E1La*	41.878	-93.0977
PI 548629	'Weber 84'	Iowa, United States	*e1-as*	*E1La*	41.878	-93.0977
PI 548679	'Mitchell'	Iowa, United States	*e1-as*	*E1La*	41.878	-93.0977
PI 548689	'B 216'	Iowa, United States	*e1-as*	*E1La*	41.878	-93.0977
PI 548690	'S 1492'	Iowa, United States	*e1-as*	*E1La*	41.878	-93.0977
PI 548538	'Columbus'	Kansas, United States	*e1-as*	*E1La*	39.0119	-98.4842
PI 548541	'Crawford'	Kansas, United States	*e1-as*	*E1La*	39.0119	-98.4842

PI 548549	'DeSoto'	Kansas, United States	*e1-as*	*E1La*	39.0119	-98.4842
PI 548555	'Douglas'	Kansas, United States	*e1-as*	*E1La*	39.0119	-98.4842
PI 548606	'Pomona'	Kansas, United States	*e1-as*	*E1La*	39.0119	-98.4842
PI 548619	'Sparks'	Kansas, United States	*e1-as*	*E1La*	39.0119	-98.4842
PI 586980	'KS3494'	Kansas, United States	*e1-as*	*E1La*	39.0119	-98.4842
PI 586981	'KS4694'	Kansas, United States	*e1-as*	*E1La*	39.0119	-98.4842
PI 515961	'Pennyrile'	Kentucky, United States	*e1-as*	*E1La*	37.8393332	-84.2700179
PI 590932	'CF461'	Kentucky, United States	*e1-as*	*E1La*	37.8393332	-84.2700179
PI 611112	'7499'	Kentucky, United States	*e1-as*	*E1La*	37.8393332	-84.2700179
PI 567178	'Black Jet'	Maine, United States	*e1-as*	*E1La*	45.253783	-69.4454689
FC 30683	NA	Manitoba, Canada	*e1-as*	*E1La*	53.7609	-98.8139
PI 548607	'Portage'	Manitoba, Canada	*e1-as*	*E1La*	53.7609	-98.8139
PI 510670	'Morgan'	Maryland, United States	*e1-as*	*E1La*	39.0457549	-76.6412712
PI 548598	'Miles'	Maryland, United States	*e1-as*	*E1La*	39.0457549	-76.6412712
PI 548633	'Wye'	Maryland, United States	*e1-as*	*E1La*	39.0457549	-76.6412712
PI 548652	'Bass'	Maryland, United States	*e1-as*	*E1La*	39.0457549	-76.6412712
PI 559931	'Corsica'	Maryland, United States	*e1-as*	*E1La*	39.0457549	-76.6412712
PI 601983	'Donegal'	Maryland, United States	*e1-as*	*E1La*	39.0457549	-76.6412712
PI 632418	'Tara'	Maryland, United States	*e1-as*	*E1La*	39.0457549	-76.6412712
PI 548673	'Ottawa'	Michigan, United States	*e1-as*	*E1La*	44.3148443	-85.6023643
PI 572244	'Dimon'	Michigan, United States	*e1-as*	*E1La*	44.3148443	-85.6023643
PI 602060	'Olympus'	Michigan, United States	*e1-as*	*E1La*	44.3148443	-85.6023643
PI 608438	'Titan'	Michigan, United States	*e1-as*	*E1La*	44.3148443	-85.6023643
PI 639693	'Skylla'	Michigan, United States	*e1-as*	*E1La*	44.3148443	-85.6023643
PI 513382	'Glenwood'	Minnesota, United States	*e1-as*	*E1La*	46.729553	-94.6858998
PI 542042	'Kato'	Minnesota, United States	*e1-as*	*E1La*	46.729553	-94.6858998
PI 542768	'Sturdy'	Minnesota, United States	*e1-as*	*E1La*	46.729553	-94.6858998
PI 542769	'Proto'	Minnesota, United States	*e1-as*	*E1La*	46.729553	-94.6858998
PI 546038	'Kasota'	Minnesota, United States	*e1-as*	*E1La*	46.729553	-94.6858998
PI 548393	'Norsoy'	Minnesota, United States	*e1-as*	*E1La*	46.729553	-94.6858998
PI 548499	'Ada'	Minnesota, United States	*e1-as*	*E1La*	46.729553	-94.6858998
PI 548500	'Swift'	Minnesota, United States	*e1-as*	*E1La*	46.729553	-94.6858998
PI 548508	'Anoka'	Minnesota, United States	*e1-as*	*E1La*	46.729553	-94.6858998
PI 548534	'Clay'	Minnesota, United States	*e1-as*	*E1La*	46.729553	-94.6858998
PI 548535	'Norman'	Minnesota, United States	*e1-as*	*E1La*	46.729553	-94.6858998
PI 548561	'Hodgson'	Minnesota, United States	*e1-as*	*E1La*	46.729553	-94.6858998
PI 548567	'Grande'	Minnesota, United States	*e1-as*	*E1La*	46.729553	-94.6858998
PI 548581	'Hodgson 78'	Minnesota, United States	*e1-as*	*E1La*	46.729553	-94.6858998
PI 548582	'McCall'	Minnesota, United States	*e1-as*	*E1La*	46.729553	-94.6858998
PI 548611	'Renville'	Minnesota, United States	*e1-as*	*E1La*	46.729553	-94.6858998
PI 548621	'Traverse'	Minnesota, United States	*e1-as*	*E1La*	46.729553	-94.6858998
PI 548668	'A-100'	Minnesota, United States	*e1-as*	*E1La*	46.729553	-94.6858998
PI 548680	'Pridesoy 57'	Minnesota, United States	*e1-as*	*E1La*	46.729553	-94.6858998
PI 557010	'Bert'	Minnesota, United States	*e1-as*	*E1La*	46.729553	-94.6858998
PI 557011	'Leslie'	Minnesota, United States	*e1-as*	*E1La*	46.729553	-94.6858998
PI 562373	'Lambert'	Minnesota, United States	*e1-as*	*E1La*	46.729553	-94.6858998
PI 564524	'Alpha'	Minnesota, United States	*e1-as*	*E1La*	46.729553	-94.6858998
PI 583364	'Faribault'	Minnesota, United States	*e1-as*	*E1La*	46.729553	-94.6858998
PI 583365	'Hendricks'	Minnesota, United States	*e1-as*	*E1La*	46.729553	-94.6858998
PI 592389	'Freeborn'	Minnesota, United States	*e1-as*	*E1La*	46.729553	-94.6858998
PI 602593	'MN1301'	Minnesota, United States	*e1-as*	*E1La*	46.729553	-94.6858998
PI 608726	'MN1401'	Minnesota, United States	*e1-as*	*E1La*	46.729553	-94.6858998
PI 612763	'MN1801'	Minnesota, United States	*e1-as*	*E1La*	46.729553	-94.6858998
PI 616498	'MN1302'	Minnesota, United States	*e1-as*	*E1La*	46.729553	-94.6858998
PI 618613	'MN0902CN'	Minnesota, United States	*e1-as*	*E1La*	46.729553	-94.6858998
PI 629004	'MN0201'	Minnesota, United States	*e1-as*	*E1La*	46.729553	-94.6858998
PI 518663	'Avery'	Missouri, United States	*e1-as*	*E1La*	37.9642529	-91.8318334
PI 543793	'Delsoy 4500'	Missouri, United States	*e1-as*	*E1La*	37.9642529	-91.8318334
PI 548546	'Custer'	Missouri, United States	*e1-as*	*E1La*	37.9642529	-91.8318334
PI 548602	'Oksoy'	Missouri, United States	*e1-as*	*E1La*	37.9642529	-91.8318334
PI 548613	'Scott'	Missouri, United States	*e1-as*	*E1La*	37.9642529	-91.8318334
PI 548670	'Cypress No. 1'	Missouri, United States	*e1-as*	*E1La*	37.9642529	-91.8318334
PI 560206	'Delsoy 4210'	Missouri, United States	*e1-as*	*E1La*	37.9642529	-91.8318334
PI 560207	'Delsoy 4710'	Missouri, United States	*e1-as*	*E1La*	37.9642529	-91.8318334
PI 578057	'Saline'	Missouri, United States	*e1-as*	*E1La*	37.9642529	-91.8318334
PI 595363	'Mustang'	Missouri, United States	*e1-as*	*E1La*	37.9642529	-91.8318334
PI 598124	'Maverick'	Missouri, United States	*e1-as*	*E1La*	37.9642529	-91.8318334
PI 548591	'Logan'	Nebraska, United States	*e1-as*	*E1La*	41.4925374	-99.9018131
PI 548597	'Mead'	Nebraska, United States	*e1-as*	*E1La*	41.4925374	-99.9018131
PI 548605	'Platte'	Nebraska, United States	*e1-as*	*E1La*	41.4925374	-99.9018131
PI 552538	'Dunbar'	Nebraska, United States	*e1-as*	*E1La*	41.4925374	-99.9018131
PI 561858	'Holt'	Nebraska, United States	*e1-as*	*E1La*	41.4925374	-99.9018131
PI 561860	'Lancaster'	Nebraska, United States	*e1-as*	*E1La*	41.4925374	-99.9018131
PI 573008	'Colfax'	Nebraska, United States	*e1-as*	*E1La*	41.4925374	-99.9018131
PI 595753	'ODell'	Nebraska, United States	*e1-as*	*E1La*	41.4925374	-99.9018131
PI 595754	'Nemaha'	Nebraska, United States	*e1-as*	*E1La*	41.4925374	-99.9018131
PI 610670	'NE3297'	Nebraska, United States	*e1-as*	*E1La*	41.4925374	-99.9018131
PI 610671	'NE3399'	Nebraska, United States	*e1-as*	*E1La*	41.4925374	-99.9018131
PI 614832	'NE3400'	Nebraska, United States	*e1-as*	*E1La*	41.4925374	-99.9018131
PI 614833	'NE1900'	Nebraska, United States	*e1-as*	*E1La*	41.4925374	-99.9018131
PI 634827	'NE2701'	Nebraska, United States	*e1-as*	*E1La*	41.4925374	-99.9018131
PI 548651	'Merrimax'	New Hampshire, United States	*e1-as*	*E1La*	43.1939	-71.5724
PI 634813	'LaMoure'	North Dakota, United States	*e1-as*	*E1La*	47.5514926	-101.002012
PI 534645	'Resnik'	Ohio, United States	*e1-as*	*E1La*	40.4173	-82.9071
PI 534646	'Flyer'	Ohio, United States	*e1-as*	*E1La*	40.4173	-82.9071
PI 534647	'GR8836'	Ohio, United States	*e1-as*	*E1La*	40.4173	-82.9071
PI 534648	'GR8936'	Ohio, United States	*e1-as*	*E1La*	40.4173	-82.9071

PI 536635	'Sprite'	Ohio, United States	*e1-as*	*E1La*	40.4173	-82.9071
PI 540551	'Hobbit'	Ohio, United States	*e1-as*	*E1La*	40.4173	-82.9071
PI 540552	'Hoyt'	Ohio, United States	*e1-as*	*E1La*	40.4173	-82.9071
PI 542709	'Hayes'	Ohio, United States	*e1-as*	*E1La*	40.4173	-82.9071
PI 542710	'Chapman'	Ohio, United States	*e1-as*	*E1La*	40.4173	-82.9071
PI 542711	'Edison'	Ohio, United States	*e1-as*	*E1La*	40.4173	-82.9071
PI 543856	'Pixie'	Ohio, United States	*e1-as*	*E1La*	40.4173	-82.9071
PI 543857	'Gnome 85'	Ohio, United States	*e1-as*	*E1La*	40.4173	-82.9071
PI 546373	'Hobbit 87'	Ohio, United States	*e1-as*	*E1La*	40.4173	-82.9071
PI 546374	'Sprite 87'	Ohio, United States	*e1-as*	*E1La*	40.4173	-82.9071
PI 546375	'Amcor 89'	Ohio, United States	*e1-as*	*E1La*	40.4173	-82.9071
PI 548505	'Amcor'	Ohio, United States	*e1-as*	*E1La*	40.4173	-82.9071
PI 548529	'Century 84'	Ohio, United States	*e1-as*	*E1La*	40.4173	-82.9071
PI 548565	'Gnome'	Ohio, United States	*e1-as*	*E1La*	40.4173	-82.9071
PI 548579	'Henry'	Ohio, United States	*e1-as*	*E1La*	40.4173	-82.9071
PI 548580	'Madison'	Ohio, United States	*e1-as*	*E1La*	40.4173	-82.9071
PI 548599	'Monroe'	Ohio, United States	*e1-as*	*E1La*	40.4173	-82.9071
PI 548612	'Ross'	Ohio, United States	*e1-as*	*E1La*	40.4173	-82.9071
PI 548614	'Sherman'	Ohio, United States	*e1-as*	*E1La*	40.4173	-82.9071
PI 548634	'Zane'	Ohio, United States	*e1-as*	*E1La*	40.4173	-82.9071
PI 561700	'Erie'	Ohio, United States	*e1-as*	*E1La*	40.4173	-82.9071
PI 564718	'Thorne'	Ohio, United States	*e1-as*	*E1La*	40.4173	-82.9071
PI 567902	'Charleston'	Ohio, United States	*e1-as*	*E1La*	40.4173	-82.9071
PI 576145	'Sandusky'	Ohio, United States	*e1-as*	*E1La*	40.4173	-82.9071
PI 576146	'Vertex'	Ohio, United States	*e1-as*	*E1La*	40.4173	-82.9071
PI 584469	'Ohio FG1'	Ohio, United States	*e1-as*	*E1La*	40.4173	-82.9071
PI 584470	'Ohio FG2'	Ohio, United States	*e1-as*	*E1La*	40.4173	-82.9071
PI 593463	'General'	Ohio, United States	*e1-as*	*E1La*	40.4173	-82.9071
PI 593654	'Stressland'	Ohio, United States	*e1-as*	*E1La*	40.4173	-82.9071
PI 595843	'Flint'	Ohio, United States	*e1-as*	*E1La*	40.4173	-82.9071
PI 596407	'Defiance'	Ohio, United States	*e1-as*	*E1La*	40.4173	-82.9071
PI 612594	'Kottman'	Ohio, United States	*e1-as*	*E1La*	40.4173	-82.9071
PI 612930	'Tiffin'	Ohio, United States	*e1-as*	*E1La*	40.4173	-82.9071
PI 612931	'HF93-083'	Ohio, United States	*e1-as*	*E1La*	40.4173	-82.9071
PI 612932	'HF93-035'	Ohio, United States	*e1-as*	*E1La*	40.4173	-82.9071
PI 614153	'Croton 3.9'	Ohio, United States	*e1-as*	*E1La*	40.4173	-82.9071
PI 614154	'Darby'	Ohio, United States	*e1-as*	*E1La*	40.4173	-82.9071
PI 614155	'HS93-4118'	Ohio, United States	*e1-as*	*E1La*	40.4173	-82.9071
PI 614806	'Troll'	Ohio, United States	*e1-as*	*E1La*	40.4173	-82.9071
PI 614807	'Stout'	Ohio, United States	*e1-as*	*E1La*	40.4173	-82.9071
PI 614808	'Strong'	Ohio, United States	*e1-as*	*E1La*	40.4173	-82.9071
PI 629008	'OHIO FG3'	Ohio, United States	*e1-as*	*E1La*	40.4173	-82.9071
PI 632401	'APEX'	Ohio, United States	*e1-as*	*E1La*	40.4173	-82.9071
PI 632402	'STALWART'	Ohio, United States	*e1-as*	*E1La*	40.4173	-82.9071
PI 633608	'Dilworth'	Ohio, United States	*e1-as*	*E1La*	40.4173	-82.9071
PI 642768	'OHIO FG5'	Ohio, United States	*e1-as*	*E1La*	40.4173	-82.9071
PI 643146	'Prohio'	Ohio, United States	*e1-as*	*E1La*	40.4173	-82.9071
PI 279648	'Hardome'	Ontario, Canada	*e1-as*	*E1La*	51.253775	-85.323214
PI 438364	'Merit'	Ontario, Canada	*e1-as*	*E1La*	51.253775	-85.323214
PI 548311	'Capital'	Ontario, Canada	*e1-as*	*E1La*	51.253775	-85.323214
PI 548539	'Comet'	Ontario, Canada	*e1-as*	*E1La*	51.253775	-85.323214
PI 548544	'Crest'	Ontario, Canada	*e1-as*	*E1La*	51.253775	-85.323214
PI 548570	'Harcor'	Ontario, Canada	*e1-as*	*E1La*	51.253775	-85.323214
PI 548573	'Harosoy'	Ontario, Canada	*e1-as*	*E1La*	51.253775	-85.323214
PI 548576	'Harwood'	Ontario, Canada	*e1-as*	*E1La*	51.253775	-85.323214
PI 548623	'Vansoy'	Ontario, Canada	*e1-as*	*E1La*	51.253775	-85.323214
PI 548641	'Haroson'	Ontario, Canada	*e1-as*	*E1La*	51.253775	-85.323214
PI 548642	'Maple Donovan'	Ontario, Canada	*e1-as*	*E1La*	51.253775	-85.323214
PI 548644	'OAC Musca'	Ontario, Canada	*e1-as*	*E1La*	51.253775	-85.323214
PI 548646	'RCAT Alliance'	Ontario, Canada	*e1-as*	*E1La*	51.253775	-85.323214
PI 548647	'RCAT Persian'	Ontario, Canada	*e1-as*	*E1La*	51.253775	-85.323214
PI 567785	'OAC Shire'	Ontario, Canada	*e1-as*	*E1La*	51.253775	-85.323214
PI 567786	'OAC Talbot'	Ontario, Canada	*e1-as*	*E1La*	51.253775	-85.323214
PI 572241	'Brock'	Ontario, Canada	*e1-as*	*E1La*	51.253775	-85.323214
PI 572243	'Harovinton'	Ontario, Canada	*e1-as*	*E1La*	51.253775	-85.323214
FC 32141	Early Sunrise	South Dakota, United States	*e1-as*	*E1La*	43.9695148	-99.9018131
PI 599299	'Stride'	South Dakota, United States	*e1-as*	*E1La*	43.9695148	-99.9018131
PI 599300	'Surge'	South Dakota, United States	*e1-as*	*E1La*	43.9695148	-99.9018131
PI 518668	'TN 4-86'	Tennessee, United States	*e1-as*	*E1La*	35.5174913	-86.5804473
PI 598222	'TN 4-94'	Tennessee, United States	*e1-as*	*E1La*	35.5174913	-86.5804473
PI 548678	'HP-963'	Texas, United States	*e1-as*	*E1La*	31.9685988	-99.9018131
PI 548355	'Kagon'	Wisconsin, United States	*e1-as*	*E1La*	43.7844	-88.7879
PI 548509	'Dunn'	Wisconsin, United States	*e1-as*	*E1La*	43.7844	-88.7879
PI 548568	'Grant'	Wisconsin, United States	*e1-as*	*E1La*	43.7844	-88.7879
PI 548601	'Norchief'	Wisconsin, United States	*e1-as*	*E1La*	43.7844	-88.7879

*Latitude and longitude coordinates for each accession were obtained from the GRIN, where available. Where unavailable, coordinates for state/province of origin were obtained from Google geocoding.
†*E1* and *E1La* alleles were estimated based on SoySNP50k proxy SNPs.

Supplemental Table 7. *E1* and *E1La* genotype status of North Dakota tofu breeding lines

Lines	*E1*	*E1La*
ND18-19466	*e1-as*	*E1La*
ND18-19469	*e1-as*	*E1La*
ND18-19470	*e1-as*	*E1La*
ND18-19471	*e1-as*	*E1La*
ND18-19485	*e1-as*	*E1La*
ND18-19486	*e1-as*	*E1La*
ND18-19494	*e1-as*	*E1La*
ND18-19497	*e1-as*	*E1La*
ND18-19515	*e1-as*	*E1La*
ND18-19516	*e1-as*	*E1La*
ND18-19538	*e1-as*	*E1La*
ND18-19544	*e1-as*	*E1La*
ND18-19549	*e1-as*	*E1La*
ND18-19551	*e1-as*	*E1La*
ND18-19552	*e1-as*	*E1La*
ND18-19553	*e1-as*	*E1La*
ND13-7510	*e1-as*	*E1La*
ND16-8257	*e1-as*	*E1La*
ND16-8305	*e1-as*	*E1La*

Supplemental Table 8. *E1* and *E1La* genotype status of North Dakota natto breeding lines

Lines	*E1*	*E1La*
ND18-17704	*e1-as*	*e1la:K82E*
ND18-17726	*e1-as*	*e1la:K82E*
ND18-17730	*e1-as*	*e1la:K82E*
ND18-17737	*e1-as*	*e1la:K82E*
ND18-17740	*e1-as*	*e1la:K82E*
ND18-17749	*e1-as*	*e1la:K82E*
ND18-17752	*e1-as*	*e1la:K82E*
ND18-17757	*e1-as*	*e1la:K82E*
ND18-17761	*e1-as*	*e1la:K82E*
ND18-17765	*e1-as*	*e1la:K82E*
ND18-17772	*e1-as*	*e1la:K82E*
ND18-17786	*e1-as*	*e1la:K82E*
ND18-17791	*e1-as*	*e1la:K82E*
ND18-17792	*e1-as*	*e1la:K82E*
ND18-17806	*e1-as*	*e1la:K82E*
ND18-17817	*e1-as*	*e1la:K82E*
ND18-17826	*e1-as*	*e1la:K82E*
ND18-17839	*e1-as*	*e1la:K82E*
ND18-19665	*e1-as*	*E1La*
ND18-19690	*e1-as*	*e1la:K82E*
ND18-19698	*e1-as*	*e1la:K82E*
ND18-19713	*e1-as*	*e1la:K82E*
ND18-19715	*e1-as*	*E1La*
ND18-19720	*e1-as*	*e1la:K82E*
ND18-19726	*e1-as*	*e1la:K82E*
ND12-20324	*e1-as*	*e1la:K82E*
